# Flexural bending of southern Tibet in a retro foreland setting

**DOI:** 10.1038/srep12076

**Published:** 2015-07-15

**Authors:** Erchie Wang, Peter J. J. Kamp, Ganqing Xu, Kip V. Hodges, Kai Meng, Lin Chen, Gang Wang, Hui Luo

**Affiliations:** 1 State Key Laboratory of Lithospheric Evolution, Institute of Geology and Geophysics, Chinese Academy of Sciences, Beijing 100029, China; 2 CAS Centre for Excellence in Tibetan Plateau Earth Sciences, Beijing 100101, China; 3 Earth Sciences, School of Science, University of Waikato, Private Bag 3105, Hamilton 2001, New Zealand; 4 School of Earth and Space Exploration, Arizona State University, Tempe, AZ85287-6004, USA; 5 College of Earth Sciences, Chengdu University of Technology, Chengdu 610059, China; 6 Nanjing Institute of Geology and Palaeontology, Chinese Academy of Sciences, Nanjing 210008, China

## Abstract

The highest elevation of the Tibetan Plateau, lying 5,700 m above sea level, occurs within the part of the Lhasa block immediately north of the India-Tibet suture zone (Yarlung Zangbo suture zone, YZSZ), being 700 m higher than the maximum elevation of more northern parts of the plateau. Various mechanisms have been proposed to explain this differentially higher topography and the rock uplift that led to it, invoking crustal compression or extension. Here we present the results of structural investigations along the length of the high elevation belt and suture zone, which rather indicate flexural bending of the southern margin of the Lhasa block (Gangdese magmatic belt) and occurrence of an adjacent foreland basin (Kailas Basin), both elements resulting from supra-crustal loading of the Lhasa block by the Zangbo Complex (Indian plate rocks) via the Great Counter Thrust. Hence we interpret the differential elevation of the southern margin of the plateau as due originally to uplift of a forebulge in a retro foreland setting modified by subsequent processes. Identification of this flexural deformation has implications for early evolution of the India-Tibet continental collision zone, implying an initial (Late Oligocene) symmetrical architecture that subsequently transitioned into the present asymmetrical wedge architecture.

Opinions regarding the origin of the differential uplift and exhumation of the Gangdese magmatic belt, compared with more northern parts of the Lhasa block (Fig. 1), are diverse and range between (i) thrusting of the southern margin of the Gangdese belt southward over Indian plate-derived rocks on an inferred Gangdese Thrust Fault^1^,^2^,^3^,^4^,^5^, and (ii) extensional or transtensional processes^6^. In contrast, we hypothesise that the assembly of structural elements within the YZSZ, including differential elevation of the Gangdese belt, results from northward thrusting of Indian plate-derived rocks upon the Lhasa block in a retro foreland setting, resulting in a foreland basin (Kailas Basin) and forebulge (elevated Gangdese belt). To test this hypothesis we investigated and mapped, along parts of the 2,000 km long YZSZ, the structural and stratigraphic relationships between the Gangdese belt, the adjacent Upper Oligocene-Lowermost Miocene Kailas Basin and northernmost units of the Indian plate (Zangbo Complex) (Fig. 2A-A”, SI1-1). Support for our hypothesis critically depends upon multiple geological events occurring concurrently: (i) uplift of the Gangdese belt, which we assess from timing of exhumation through fission track thermo-chronology, (ii) timing of sedimentation in the Kailas Basin, assessed from U-Pb geochronology of tuff low in the succession, and (iii), timing of thrust emplacement of the Zangbo Complex upon the Kailas succession via the Great Counter Thrust (GCT), which we assess from field petrography and radiolarian content of Kailas conglomerate clasts. The same timing of these events would point to their assembly within the suture zone in a retro foreland system; that is, advance of a fold-thrust belt (Zangbo Complex) towards hinterland lithosphere (Lhasa block) thereby loading it to form a foreland basin (Kailas Basin) and associated forebulge (Gangdese belt).

**Geological setting**

Our study region is located at the juxtaposition of continental crust forming southern Tibet (Lhasa block) and the Tethyan Himalaya along the YZSZ (Fig. 1). Between them lies (i) the Zangbo Complex, comprising elements of a Cretaceous subduction wedge (Xigaze flysch) with ophiolite that accreted to the leading edge of the overriding plate (Tibet) outboard of the Lhasa block during intra-oceanic subduction prior to the Tertiary start of continent-continent collision, and (ii), a much younger (U. Oligocene) sedimentary succession (Kailas Basin) that onlaps the Gangdese belt and is separated from the Zangbo Complex by the GCT (Fig. 2A-A”).

## Results

### Asymmetrical bending of the Lhasa block

**Figure 1:**
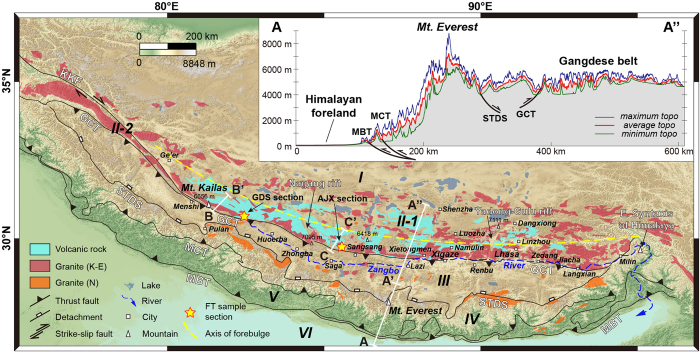
Generalized geologic and geomorphic map of the southern margin of the Tibetan plateau showing a NE-SW topographic profile (A-A”) in the Xigaze area (Geological units were modified from The Geology Map of Tibetan Plateau and Adjacent Regions [1:1500000]. Digital elevation data were downloaded from the website of http://srtm.csi.cgiar.org. Map was drawn by K. M. using the software of Adobe Illustrator 18.0.0).

**Figure 2:**
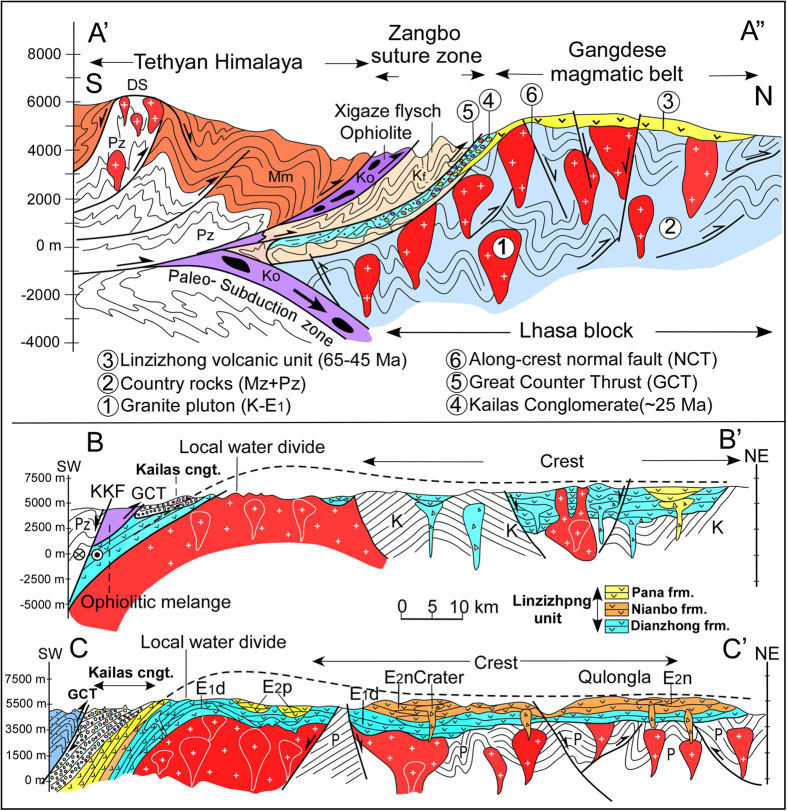
Generalized geological cross-section (A’-A”) between the Tethyan Himalayas and the Gangdese magmatic belt (southernmost Tibet) in the Xigaze area, showing the tectonic units comprising the Gangdese belt and Zangbo Complex. Geological cross-section **B-B’** in the Kailas area (western Gangdese belt), showing asymmetric bending. Cross-section **C-C’** in the Saga area (mid-Gangdese belt), also showing asymmetric bending of the Linzizhong unit and underlying basement.

The crystalline and sedimentary basement forming the Lhasa block is mostly of Late Palaeozoic-Mesozoic age^7^. Extensive granitoid intrusions, collectively known as the Gangdese belt, developed as a Cretaceous-Early Cenozoic Andean-type arc^8^,^9^, extending for 2,000 km upon and within the southern margin of the Lhasa block (Fig. 1, SI1-1). A volcanic succession of calc-alkaline lavas and related sedimentary facies (Linzizhong Group, LZ) with 65–45 Ma ages^10^ overlies basement, separated from it by a regional unconformity, forming a useful structural marker (Figs 1 and 2). Along the crest of the Gangdese belt and north of it, the LZ succession is mostly sub-horizontal (Fig. 2B-B’, C-C’, SI1–2a–c, 1-3a–c), whereas along the sharp southern margin of the belt the LZ unit curves over, dipping increasingly more steeply to the south (SI1–4a–b). Hence regional changes in dip of the LZ volcanic rocks help define a first-order asymmetric fold with a sub-horizontal fold axis across the Gangdese belt.

### Timing of exhumation of the Gangdese batholith

We have applied fission track analysis to multiple samples from each of two vertical transects to establish the timing and duration of rock uplift and exhumation of the Gangdese belt. Seven samples were collected from the Napijia (NPJ) section east of Mt. Kailas at elevations ranging between 5,100 and 5,750 m and three samples were collected from the Ajuexiong (AJX) section west of Sangsang at elevations of 5,150–5,500 m (Fig. 1, SI2-1). Our analysis yielded mean apatite fission track ages of 16.5 to 23.4 Ma and 17.9 to 21.4 Ma for these two sections, respectively, with long mean track lengths mostly in the range14.5 to 15.5 microns (Table SI2-1-2). These data suggest rapid cooling of the sample host rocks through a partial annealing zone, although final exhumation to the surface probably occurred later^5^. Hence the measured ages can be interpreted as giving minimum ages of the timing of the start of exhumation (L. Oligocene) and its duration, which was probably driven by rock uplift. These data are consistent with other apatite fission track data reported for the Gangdese belt^2^,^5^,^11^,^12^,^13^. Zircon fission track data for two of our samples yield Eocene ages (Table SI2-1-1), which are interpreted as late stage batholith cooling to ambient crustal temperatures well before later exhumation cooling recorded by the apatite fission track data.

### Kailas foreland basin: Stratigraphic age and provenance

The Kailas Basin flanks the southern margin of the Gangdese belt, having a strike length of ~2,000 km (SI1-1). Our own and prior investigations^6^ show that the Kailas succession unconformably onlaps basement of the Gangdese belt (SI1–5a–c), and hence the basin clearly formed upon the Lhasa block (Fig. 2A-A”). The sediments dip to the south mainly at high angles (>40°) so minimum width of the original basin succession is exposed, the maximum preserved thickness being 2,000 m in the Mt. Kailas area where the dip is uncharacteristically shallow at 10 degrees (SI1–6). The succession can contain crystal tuff or trachy-andesite, with eruptive ages of 26–24 Ma^6^ or 26–21 Ma^14^. We have mapped a 100 m-thick layer of crystal tuff near the base of the succession (SI1–7) north of Rengbu, which gave a U-Pb zircon age of 22.34 ± 0.22 Ma (Fig. 1 for location of samples, SI2-2). Sedimentary onlap of the paleo-slope surface across the Lhasa block will likely be diachronous, reported sample ages relating to different positions on the paleo-slope, indicating that sediments accumulation occurred during the L. Oligocene to earliest Miocene. Detrital zircon numerical ages of 24 Ma for the uppermost part of the succession west of Mt. Kailas, and 21 Ma at Geydo northwest of Xigaze have been reported^6^,^14^, which suggests that the succession that remains, accumulated quickly before it was structurally truncated by the GCT. There is however some evidence for syn-sedimentary deformation of the Kailas Basin margin fill. For example, at sites northeast of Menshi, 15 km west of Mt. Kailas (SI1–8a–b), there is up-section decrease in dip indicative of growth strata due to syn-sedimentary tilting across the basin margin.

The Kailas Basin succession was sourced from both the Lhasa block and the Zangbo Complex (fold-thrust belt). The basal part of the Kailas unit in the middle and eastern parts of the Gangdese belt is comprised of breccia and conglomerate, passing up into ripple cross-bedded sandstone and shale^5^,^6^,^15^,^16^,^17^. The volcanic clasts, similar in lithology and colour to the LZ volcanic unit, together with granite clasts, are likely derived from the Gangdese belt (SI1-9a–b). Chert and ophiolite clasts were derived from the Zangbo Complex (next section).

### Timing of displacement on The Great Counter Thrust

The GCT is a well-defined structure^1^,^2^,^3^,^4^,^18^,^19^,^20^,^21^,^22^,^23^, traceable along most of the entire southern edge of the Gangdese belt, dipping to the south at a mean angle of 55° (average of 40 measurements) (SI1-1). This fault juxtaposes Kailas succession and Gangdese belt rocks in the footwall with older rocks of various ages in the hanging wall. The GCT demonstrates clear evidence for top-to-north displacement^10^,^11^ (SI1–6b, 9a, c, 10a, 11a–b and 12). The difference between the average dip of the Kailas succession (>40°) compared with that of the GCT (55°) indicates that the GCT was a low angle thrust (average 15°).

The age of initiation of the GCT has not previously been tightly constrained. Limited thermo-chronology ages have been interpreted to indicate activity on the GCT, ranging from 25–10 Ma in the Zedang area^3^, 17.5 Ma in the Renbu area^22^, to 15–11 Ma in the Langxian area^21^. We have observed in many places (Sangsang, Xigaze, Zedang, Jiacha and Langxian) that the lower part of the Kailas succession contains numerous clasts identical in lithology to the ophiolite suite within the Zangbo suture zone complex—purple coloured chert and mafic and ultra-mafic clasts (SI1–10a–b, 11c). These ophiolite-derived and associated clasts are particularly evident in the lower parts of Kailas beds exposed in an area 60 ×3 km^2^ northwest of Xigaze City along the southern side of Zangbo River (SI1–13). The chert clasts contain radiolarians aged Late Jurassic to Cretaceous, the same ages as radiolarians in the ophiolite suite (SI1–14, 3–1). In addition, the dipping direction of many flat pebbles made of the radiolarian chert also indicates that these clasts were transported from the south (SI1–10b). The derivation of clasts in the footwall of the GCF (and low in the Kailas Basin succession) from rock sequences in the hanging wall (Zangbo Complex) of the GCT implies very early (c. 25–23 Ma) north-directed thrust movement on the GCT (SI1–14). These relationships further suggest that the Kailas Basin was probably short-lived, as the sedimentary succession relatively quickly became overthrust by the Zangbo Complex.

### A retro foreland setting for early continent-continent collision

**Figure 3:**
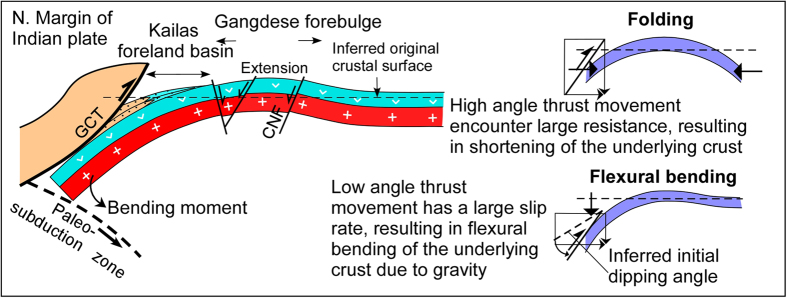
Two-dimensional elastic-bending model of the southern margin of the Lhasa block showing concurrent uplift of the Gangdese magmatic belt as a forebulge coupled with subsidence of a foreland basin in response to the tectonic load of the Zangbo Complex. Rapid L. Oligocene and E. Miocene uplift and erosion of the forebulge was concurrent with sedimentation in the Kailas foreland basin and thrust emplacement of the Zangbo Complex on the Great Counter Thrust (GCT). The inset schematic illustrates the possible thin elastic plate bending mechanisms for the region, the lower one being preferred.

We hypothesised above that the assembly of structural elements within the YZSZ resulted from thrusting of the Zangbo Complex upon the Lhasa block in a retro foreland setting, resulting in a foreland basin (Kailas Basin) and forebulge (Gangdese belt) (Fig. 3). Validation of this model would require the same timing of thrusting on the GCT, subsidence and sedimentation in the Kailas Basin and uplift of the Gangdese belt. Our results show this to be the case: sedimentation in Kailas Basin started during 26–23 Ma; displacement on the GCT was concurrent with early basin sedimentation (L. Oligocene-earliest Miocene) starting at about 25 Ma as distinctive radiolarian chert and ophiolite clasts sourced from the Zangbo Complex occur in lower parts of the succession and become more common up section, indicating that material was being shed off the front of the fold-thrust belt as it advanced towards the hinterland; and reset apatite fission track ages for a suite of vertical samples from two geographically separated sections in the Gangdese belt indicate cooling started at c. 23 Ma or a few million years earlier, which we interpret as timing the start of uplift and exhumation of the Gangdese belt. We have found no evidence for a north dipping Gangdese Thrust along the margin^1^ nor within the so-called Zedang Window^4^ having remapped that area (SI1–15–17), in support of similar observations and conclusions made by others^6^,^24^,^25^,^26^. Margin-parallel extension we have mapped as the Crest Normal Fault Zone (Fig. 2A-A”, SI1-1), particularly in areas around Nanmulin and Lhasa (SI1-19–21), is attributed here to bending along the crest of the forebulge in a retro foreland setting under supra-crustal loading (Fig. 3), rather than indicating an inferred short-lived interval of extension following slab break-off^6^,^14^ or crustal shortening (Fig. 2B).

### Flexural bending

**Figure 4:**
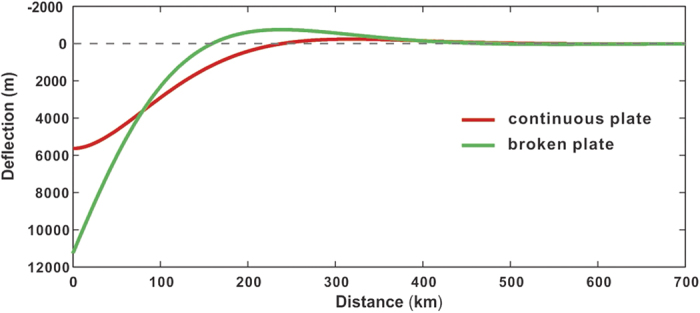
Results of numerical modelling of flexure appropriate to the northern margin of the Lhasa Terrane for both a continuous plate and a broken plate. A broken plate model simulates the retro foreland setting in the vicinity of the Yarlung Zangbo suture zone whereas a continuous plate does not.

Retro foreland settings are characterised by lithospheric flexure. Here we model the flexure appropriate to the structure of the Zangbo suture zone (Figs 3 and 4). The characteristic deflection of a loaded plate is dependent on both magnitude of the applied load and mechanical properties of the plate itself. Flexural rigidity *D* controls the wavelength of deflection, but whether the loaded plate is continuous or broken is vital to determination of the geometry of the deflection. Considering that the Zangbo suture zone is the southern edge of the Lhasa Terrane, a broken elastic plate is likely to be a better proxy for this region than a continuous plate^27^.

For a broken plate with *D* = 1.68 × 10^23^ Nm (equivalent to *Te* = 30 km, where *Te* is the effective elastic thickness of the plate), the forebulge produced by a vertically acting load of 7.28 × 10^12 ^kg.s^−2^ (equivalent to a load with a half-width of 70 km, height of 4 km and density of 2,650 kg/m^3^) results in a maximum positive deflection (height) of 755 m with a half-width of 80 km. Using the same load and the same material properties of a loaded continuous plate, the forebulge height is only 243 m. A relatively low value of *Te* = 30 km is consistent with several previous estimates for the Tibetan-Himalayan region^28^,^29^,^30^. Thus, it is plausible for a weak and broken plate loaded by a large magnitude overthrust to produce a forebulge as high as over 700 m.

The focus of this study has been on the geological elements in the Zangbo suture zone and a retro foreland explanation of their juxtaposition during the L. Oligocene and E. Miocene. The flexural model for a broken plate simulates a forebulge sufficiently high (750 m) to initiate a phase of exhumation of underlying rocks, which we have identified from our thermochronology data, which is consistent with comparable regional data sets^5^. This preferred flexural model simulates steeper dips on an unconformity surfaceflanking the forebulge than would be expected for a continuous plate (Fig. 4). The exposed width of the Kailas Basin (foredeep) is 15 km, when modelling would predict about 80 km. However the southern margin of Kailas Basin is buried beneath the Zangbo Complex and its original width is presently unknown.

Since the Early Miocene, the Zangbo suture zone and associated elements of the retro foreland system described here, have been uplifted and additional load has resulted from the emplacement of the Tethyan Himalaya rock sequences against the Zangbo suture zone complex. This load may have increased deflection of the lithosphere and resulted in a second Miocene-Pliocene phase of forebulge uplift that resulted in additional exhumation^5^. This may explain why the present elevation of the axis of the Gangdese belt, averaging 5,700 m is some 700 m above the elevation of the Tibetan Plateau to the north (Figs 1 and 2A-A”).

Our proposed model requires an orogenic belt of a high elevation and large mass that would have developed along the Himalayan Counter thrust system, so, where is it? The Zangbo River runs eastward through the Zangbo suture zone along a wide valley. We note that where the Zangbo suture zone is not cut by the Zangbo River, the geomorphology show very high topography ~6,000 m, e.g. in the area 10 km northwest of Saga. By inference, therefore, prior to the incision of the Zangbo River drainage system, the Zangbo suture zone on the hanging wall of the Great Counter thrust fault must have been an orogenic belt with high topography.

## Discussion

### Retro foreland versus extensional basin setting

We have inferred a retro foreland setting for the Zangbo suture zone, the Kailas Basin being the foredeep part of the setting. The Kailas Basin has previously been interpreted as an extensional or transtensional basin^6^,^14^. Accumulation in a contractional tectonic regime has been discounted^6^ as (i) Kailas Formation onlap of Gangdese arc basement is not typical of facies generated from the hanging walls of bounding thrust belts, (ii) there is no evidence for contractional growth structures, (iii) the lithofacies pattern is not typical of wedge-top or proximal foredeep settings, and (iv), basaltic andesites and adakitic tuff in Kailas Formation suggests a thermal pulse possibly consistent with an extensional setting, perhaps associated with slab break-off.

In proposing a retro foreland setting, we draw attention to the elements of the whole system: the fore bulge being the elevated Gangdese belt, the foredeep being the Kailas Basin, and the fold-thrust belt being the Zangbo Complex and GCT. Our focus is broader than just the basin, we are very clear about the polarity of the system and would not be expecting the Gangdese belt to be the thrust belt side of it –compare with point (i) above. Contractional growth structures within Kailas Formation are not evident but the formation is overlain by a fold-thrust belt (Zangbo Complex) that actively shed distinctive detritus into the basin ahead of its advance, and hence thrust faulting was concurrent with sedimentation-compare with point (ii) above. The exposed part of the basin lay adjacent to the forebulge (not wedge-top or proximal foredeep) and for a wholly non-marine succession the facies are going to be atypical of most foreland basins –compare with point (iii) above. Basaltic andesites and adakitic tuff are not diagnostic of extensional settings^6^ – compare with point (iv) above. Normal faults bounding a supposed extensional Kailas Basin have not been identified and neither have transtensional structures.

The 2,000 km strike length of key elements along the margin – the elevated Gangdese belt, Kailas Basin and GCT, all located within a convergent continent-continent collision zone, needs to be appropriately weighted in any tectonic interpretation of their origin. A retro foreland setting is logically the default interpretation^16^.

### Implications for evolution of the Himilaya mountain chain

**Figure 5:**
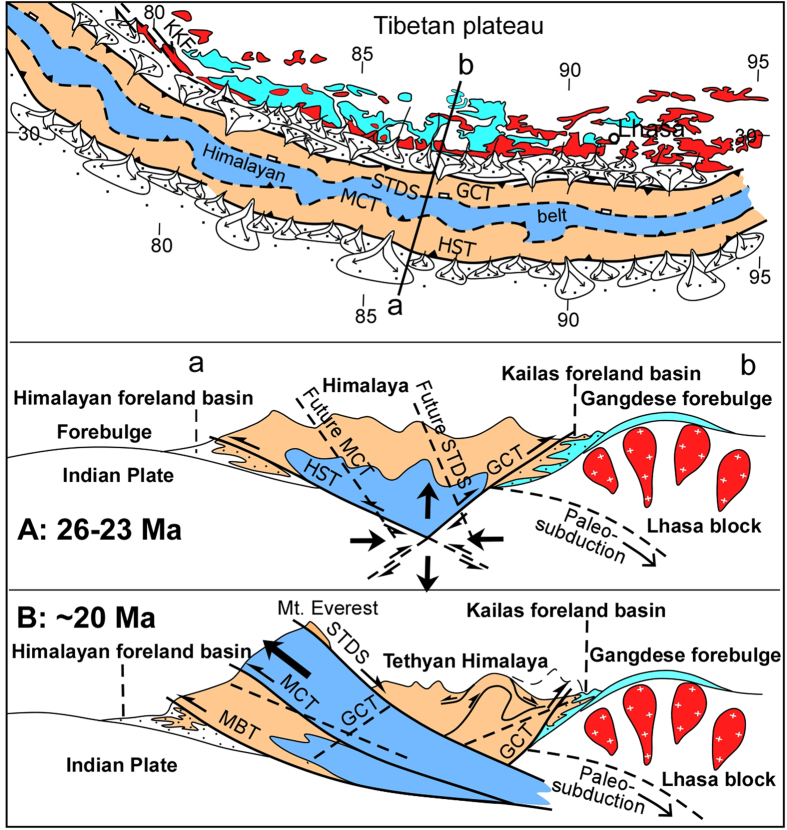
Map and cross-sections schematically illustrating a proposed two-stage tectonic development of the Himalaya orogen: **A**: (26–23 Ma) displacement on conjugated thrusts builds a central mountain belt, which loads and flexes continental crust of India and southern Tibet (Gangdese belt) forming paired foreland basins – a symmetrical architecture typical of most collisional orogens. **B**: (Neogene) continuing continent-continent shortening transitions the orogen into an asymmetrical architecture with a structurally imbricated wedge, the uppermost part (Tethyan Himalaya) collapsing northward on the South Tibet Detachment System (STDS), accentuating the bending of the Gangdese belt. Abbreviations: MCT, Main Central Thrust; GCT, Great Counter Thrust; MBT, Main Boundary Thrust; HST, Himalayan Sole Thrust.

The southerly dip of the GCT and its initial displacement during the L. Oligocene suggests that it originated as a backthrust, representing advance of a fold-thrust belt into a foreland basin. In our view, the GCT was likely conjugated with south-directed thrust faults, the Himalayan Sole Thrust (HST), to the south of the Himalayan crest at the base of the Himalayan pro-wedge, thereby building an early (latest Oligocene) symmetrical Himalaya orogeny (Fig. 5A). With inferred displacement on conjugated thrusts, crustal flexure and foreland basins developed on both the southern and northern margins of the orogen in response to the tectonic load arising from early growth of the Himalayan mountain chain. In response to continuing continent-continent collision, the architecture of the orogen transitioned from a symmetrical form with inward facing flexures to an asymmetrical form characterized by the Early Miocene through Neogene development of a structurally imbricated wedge (Fig. 5B). The MCT and STDS may have formed during this second stage of orogeny development at or soon after 22–20 Ma (SI1-18), thereby accommodating the south-directed tectonic extrusion of crystalline basement forming the High Himalaya^31^,^32^, and resulting in emplacement of Tethyan Himalaya rock sequences against the Zangbo suture zone complex, which accentuated the bending of the northern margin of the Lhasa block. These processes, together with wholesale tectonic emplacement of India crust beneath the Lhasa block, lifted the whole of the Tethyan Himalaya, the Kailas Basin and the Gangdese belt, thereby stair-casing the two foreland basins and their flexures across the collision zone to the north.

## Methods

### Fission track method

Sample preparation and experimental methods used in this study follow those reported by *Green* (1985)^33^ and *Gleadow et al.* (1986)^34^, as adopted in the University of Waikato Fission Track laboratory^35^,^36^. Apatite and zircon concentrates were separated from 2 kg samples of basement rocks using standard magnetic and heavy liquid techniques. The external detector method^37^ has been used exclusively in this study. Teflon zircon mounts were etched in NaOH:KOH eutectic solution at 230 ± 1 °C between 10 and 18 hours. Apatite and zircon mounts were irradiated in the ANSTO reactor at Lucas Heights, Sydney, Australia, with nominal fluences of 1 × 10^16^ n/cm^2^ for apatite and 2 × 10^15 ^cm2 for zircon. The fission track ages were determined using the zeta calibration method^33^,^38^ and calculated as central ages^39^. Confined track lengths in apatite were measured using a digitizing tablet connected to a computer, superimposed on the microscope field of view via a projection tube. This system was calibrated against a stage graticule ruled in 2microns divisions. Tracks with this system can be measured with a precision of 0.2 microns. Tracks were measured using the recommendations of *Laslett et al.* (1982)^40^.

### U-Pb Analytical method

Measurements of U, Th and Pb isotopes were conducted using a Cameca IMS-1280 SIMS at the Institute of Geology and Geophysics, Chinese Academy of Sciences, Beijing, China. The instrument description and analytical procedure can be found in *Li et al.* (2009)^41^. The primary O^2–^ ion beam spot was about 20–30 micron in diameter. Positive secondary ions were extracted with a 10 KV potential. Oxygen flooding was used to increase the O_2_ pressure to ca. 5 × 10^−6^ Torr in the sample chamber, enhancing the secondary Pb^+^ sensitivity to a value of 25 cps/nA/ppm for zircon. In the secondary ion beam optics, a 60 eV energy window was used, together with a mass resolution of ca. 5,400 (at 10% peak height) to separate Pb^+^ peaks from isobaric interferences. A single electron multiplier was used in ion-counting mode to measure secondary ion beam intensities by peak jumping mode. Analyses of the standard zircon TEMORA 2 were interspersed with unknown grains. Each measurement consists of 7 cycles. Pb/U calibration was performed relative to zircon standard TEMORA 2 (^206^Pb/^238^U age = 417 Ma)^42^; U and Th concentrations were calibrated against zircon standard 91500 (Th = 29 ppm, and U = 81 ppm)^43^. A long-term uncertainty of 1.5% (1 RSD) for ^206^Pb/^238^U measurements of the standard zircons was propagated to the unknowns^44^, despite that the measured ^206^Pb/^238^U error in a specific session is generally 1% (1 RSD). Measured compositions were corrected for common Pb using non-radiogenic ^204^Pb. Corrections are sufficiently small to be insensitive to the choice of common Pb composition, and an average of present-day crustal composition^46^ is used for the common Pb assuming that the common Pb is largely surface contamination introduced during sample preparation. Data reduction was carried out using the Isoplot/Ex v. 2.49 program^46^. Uncertainties on individual analyses in data tables are reported at 1σ level; Concordia U-Pb ages are quoted with 95% confidence interval, except where noted otherwise.

To monitor the external uncertainties of SIMS U-Pb zircon dating calibrated against TEMORA 2 standard, an in-house zircon standard Qinghu was alternately analysed as an unknown together with other unknown zircons. Six measurements on Qinghu zircon (see Table S2-2–1) yield a Concordia age of 159.3 ± 1.9 Ma, which is identical within error with the recommended value of 159.5 ± 0.2 Ma^47^.

### Formula used to calculate deflection

For a continuous plate, the deflection produced by a vertically acting load *V*_0_, emplaced at x = 0, is given by the following equation^48^.


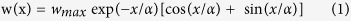


where the flexural parameter α is given by


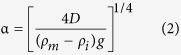


and the maximum deflection in terms of the vertical load is


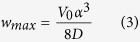


In the above equations, w is the deflection, *D* is the flexural rigidity, *ρ*_*m*_ is mantle density, *ρ*_*i*_ is load density, and g is gravity acceleration. For the broken plate, the deflection is given by
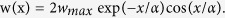


## Additional Information

**How to cite this article**: Wang, E. *et al.* Flexural bending of southern Tibet in a retro foreland setting. *Sci. Rep.* **5**, 12076; doi: 10.1038/srep12076 (2015).

## Supplementary Material

Supplementary Information
