# Supplementary material for: Flexural bending of southern Tibet in a retro foreland setting
Source: Sci Rep. 2015 Jul 15;5:12076. doi: 10.1038/srep12076 (PMC4502525; doi:10.1038/srep12076)
Supplement: Supplementary Information [file srep12076-s1.doc]

**Title:** Flexural bending of southern Tibet in a retro foreland setting

**Author list:**

1. Erchie Wang,

State Key Laboratory of Lithospheric Evolution, Institute of Geology and Geophysics, Chinese Academy of Sciences, Beijing 100029, PRC

CAS Centre for Excellence in Tibetan Plateau Earth Sciences, Beijing 100101, China

[Erchie-wang@mail.iggcas.ac.cn](mailto:Erchie-wang@mail.iggcas.ac.cn)

1. Peter J. J. Kamp,

School of Science, University of Waikato, Private Bag 3105, Hamilton 2001, NZ.

[kamp@xtra.co.nz](mailto:kamp@xtra.co.nz)

1. Gangqing Xu,

School of Science, University of Waikato, Private Bag 3105, Hamilton 2001, NZ.

[xu1@waikato.ac.nz](mailto:xu1@waikato.ac.nz)

1. Kip V. Hodges,

School of Earth and Space Exploration, Arizona State University, Tempe, AZ85287-6004, USA.

[kvhodges@asu.edu](mailto:kvhodges@asu.edu)

1. Kai Meng,

State Key Laboratory of Lithospheric Evolution, Institute of Geology and Geophysics, Chinese Academy of Sciences, Beijing 100029, PRC

[Michael.meng@mail.iggcas.ac.cn](mailto:Michael.meng@mail.iggcas.ac.cn)

1. Lin Chen,

State Key Laboratory of Lithospheric Evolution, Institute of Geology and Geophysics, Chinese Academy of Sciences, Beijing 100029, PRC

[chenlin@mail.iggcas.ac.cn](mailto:chenlin@mail.iggcas.ac.cn)

1. Gang Wang,

College of Earth Sciences, Chengdu University of Technology, Chengdu 610059, PRC

[wanggang07@cdut.cn](mailto:wanggang07@cdut.cn)

1. Hui Luo

Nanjing Institute of Geology and Palaeontology, Chinese Academy of Sciences, Nanjing 210008, PRC.

[huiluo@nigpas.ac.cn](mailto:huiluo@nigpas.ac.cn)

**SUPPLEMENTARY INFORMATION**

**Contents**

**1. p. 1-16: Geological maps and field photos;**

**2. p. 17-21: Results of fission track analysis;**

**3. p. 22-25: U-Pb Analytical results;**

**4. p. 26-27: Results of age dating of radiolarian fossils.**

**
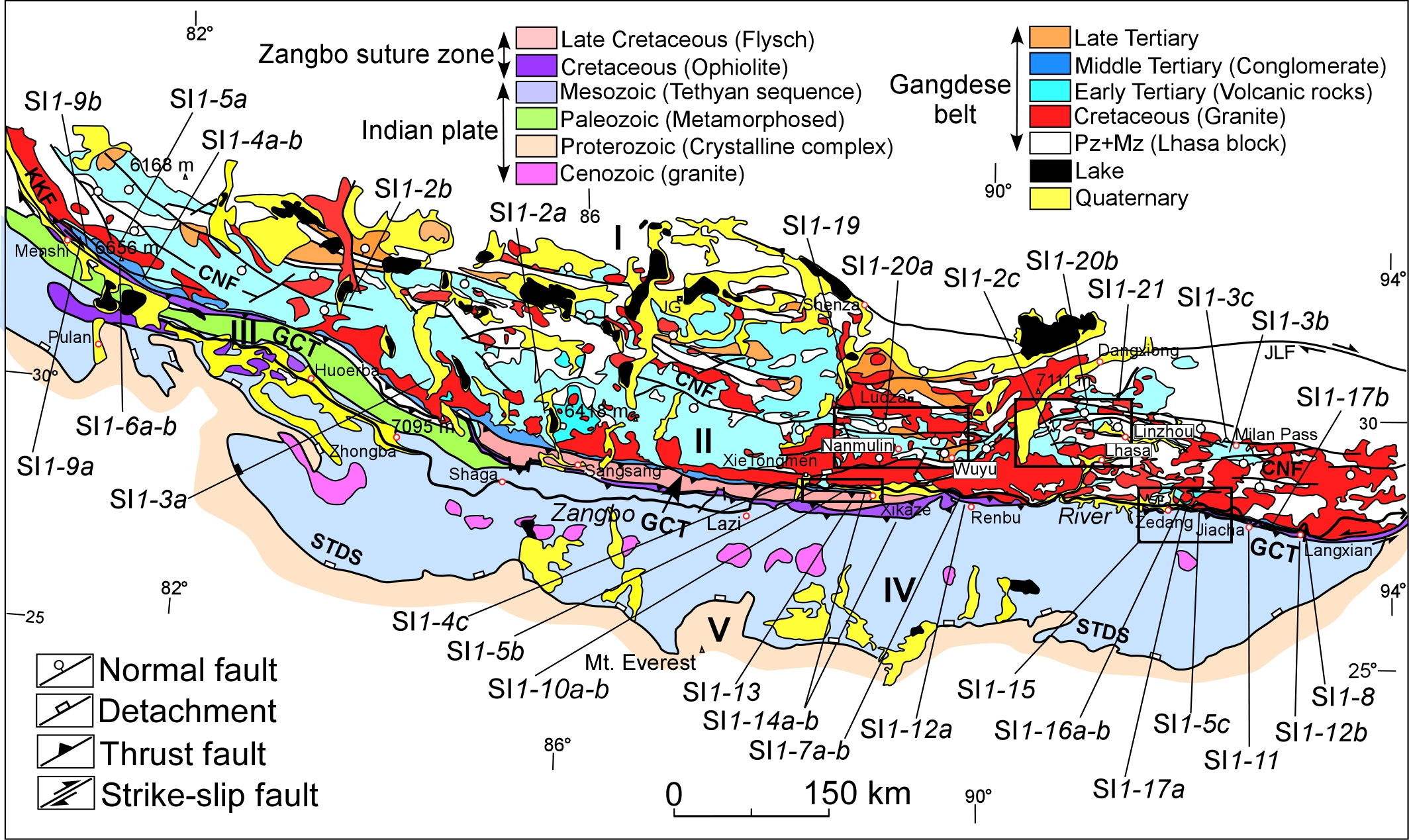
**

**SI*1-1*** Geologic map of the Gangdese magmatic belt and its adjacent areas, with the location of all of the figures in this study shown by numbers. I, Tibetan plateau; II, Gangdese magmagic belt; III, Zhongba terrane; IV, Tethyan Himalaya; V, High Himalaya; KKF, Karakorum Fault; JLF, Jiali Fault; STDS, South Tibet Detachment System; GCT, Great Counter Thrust; CNF, Crest Normal Fault.


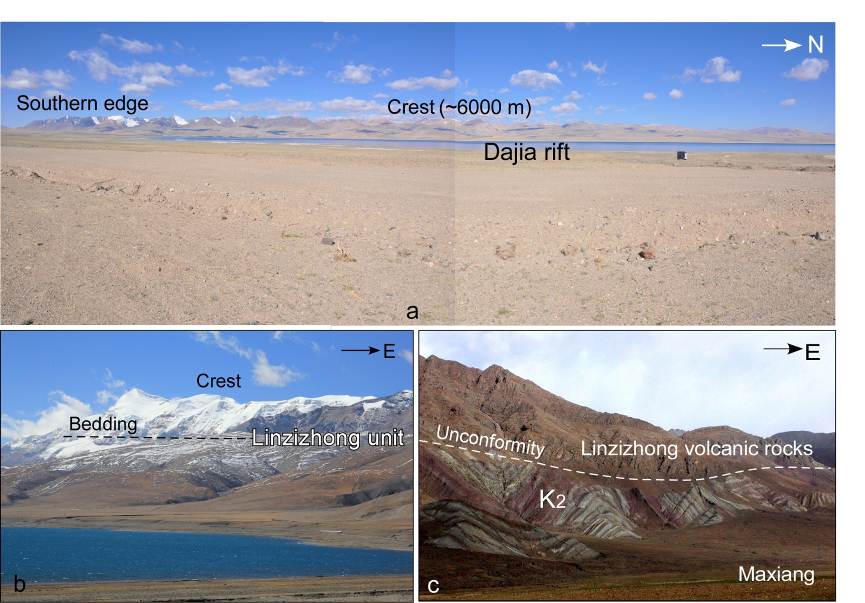


**SI*1-2*** a: The north-trending Nagang rift in an area 60 km northwest of Sangsang provides a profile view (looking west) of the plateau-like crest of the middle part of the Gangdese belt, with mean elevation ~6000 m; b: A north-trending rift in an area 60 km northeast of Huoerba provides a profile view to the north of the sub-horizontal Linzizhong volcanic rocks which hold up the plateau-like crest in the western part of the Gangdese belt. The volcanic rocks gently dip to the east along the east boundary fault of the rift; c: View looking to the northwest, of the sub-horizontal unconformity characterized by gentle relief, separating the Linzizhong volcanic unit from the folded Cretaceous red beds below it, in Maxiang, 20 km northwest of Lhasa. Note that the individual volcanic layers are not flat (white line) as their attitude reflects the relief on the unconformity surface. (Photo by E. Wang)


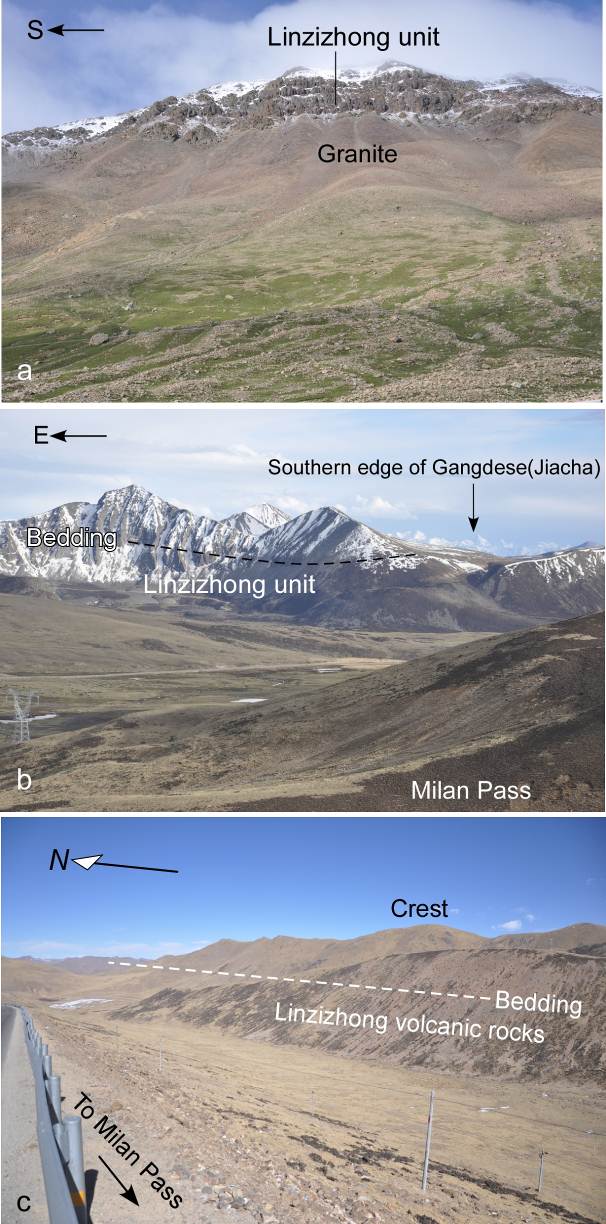


**SI*1-3*** a: View to the east of the sub-horizontal Linzizhong volcanic unit overlying granite exposed along the western edge of the Nagang rift, 65 km northeast of Zhongba; b: View from Milan Pass across the crest of the eastern part of the Gangdese belt, looking south at the sub-horizontal Linzizhong volcanic unit 5 km southwest of Songduo. The sharp peaks on the far horizon capped by snow are underlain by Kailas conglomerate that flanks the Gangdese belt on its southern margin in the Jiacha area; c: View northward in the area north of Milan Pass of sub-horizontal Linzizhong volcanic rocks, which underlie to the north the flat crest of the Gangdese belt. (Photo by E. Wang)


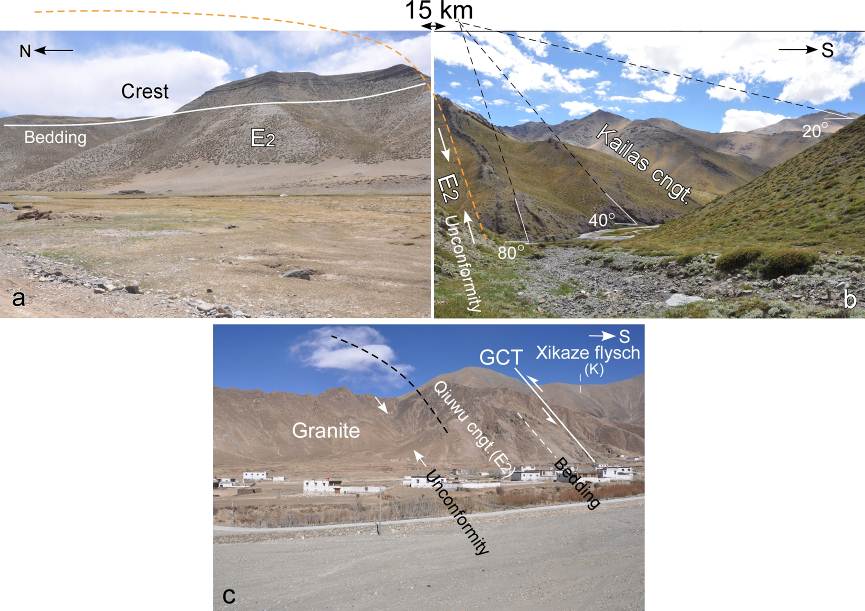


**SI*1-4*** a, b: View north of flat-lying Qiuwu conglomerate (E_2_), contemporary deposits of the Linzizhong unit, which hold up the flat crest of the western part of the Gangdese magmatic belt, in an area 15 km east of Mt. Kailas. Towards the southern margin, both the Qiuwu conglomerate and the overlying Kailas conglomerate consistently curve to the south giving rise to more rugged topography, implying that the dip formed during or after deposition of the Kailas conglomerate. Along the southern edge of the belt, Kailas conglomerate is separated from an overlying mélange complex (Zangbo suture zone) by the Great Counter Thrust (GCT), which dips to the south. The Kailas conglomerate, unlike the underlying Qiuwu unit, has progressive up-section decrease in dip (80-20°), indicative of growth strata. c: View east of the Qiuwu coal-bearing unit of Eocene age, a sedimentary sequence closely related to the Linzizhong unit, tilted to the south 15 km north of Lazi in the middle part of the Gangdese belt. It unconformably rests on granite towards the north and is structurally overlain by Xigaze flysch of Cretaceous age along the GCT. (Photo by E. Wang)


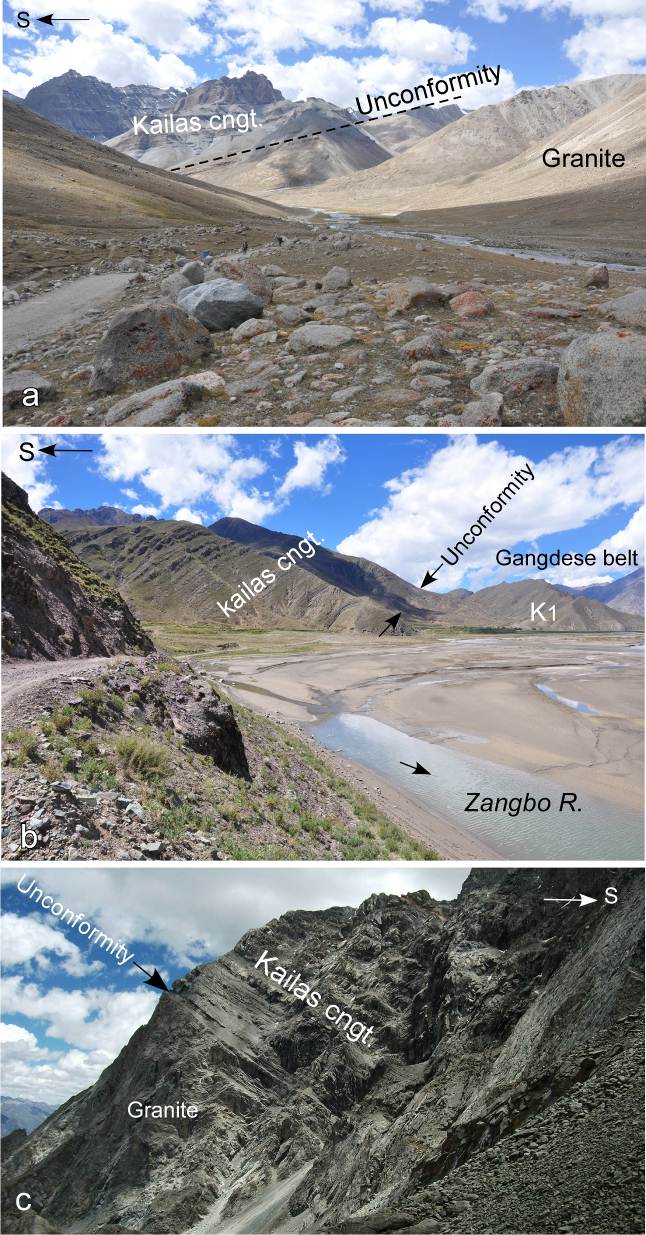


**SI*1-5*** a: View west at the lower part of Kailas conglomerate unconformably overlying granite of the Gangdese belt along the southern flank of Mt Kailas; b View west of the lower part of Kailas Conglomerate onlapping Cretaceous igneous rocks of the Gangdese belt, in an area 40 km northwest of Xigaze City. The Kailas sequence has a cyclic facies pattern, composed of alternating sandstone, mudstone and conglomerate, the latter composed mostly of well sorted and rounded clasts of purple-colored and greenish radiolarian chert similar to those in **SI*1-13b***. See **SI*1-16*** for the location; c: View east at Kailas conglomerate underlain by a granite pluton of Cretaceous age in the Luobusha area, 20 km east of Zedang. At this site Kailas conglomerate clasts are entirely composed of granite (see **SI*1-16*** for site location). (Photo by E. Wang)


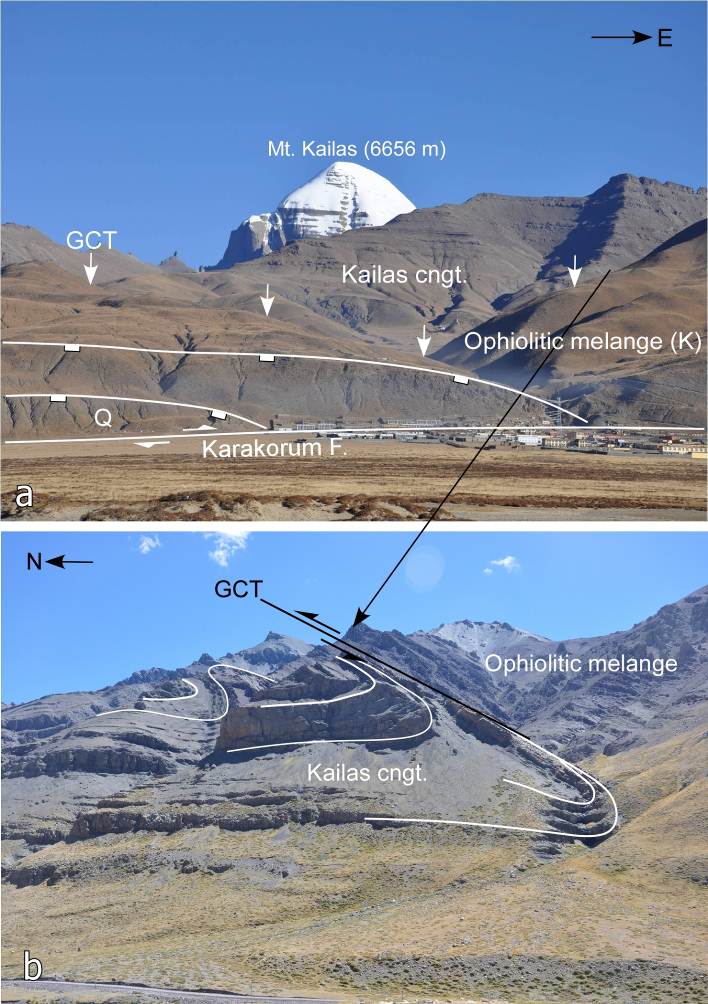


**SI*1-6*** a: View north to Mt. Kailas (6656 m), the type section for the Kailas conglomerate unit, which has an unusually low dip (5-10°) in this part of the Gangdese magmatic belt. The Kailas conglomerate is overlain to the south by ophiolitic mélange of Cretaceous age, having been emplaced by displacement on the GCT. The trace of the GCT is indicated by vertical arrows in the upper photograph. The ophiolitc mélange is truncated by the Karakorum Fault (KKF) along the southern edge of Mt. Kailas. This fault is characterized by having an active normal slip component. In becoming the footwall of the KKF, the Kailas unit was tilted to the north resulting in a decrease in dip**;** b: View northeast to the GCT along the southern margin of the Gangdese belt. The thrust here divides the ophiolitic mélange of the Zangbo suture in the hanging wall from Kailas Conglomerate in the footwall, the latter having been shortened within a large syncline overturned to the north. This fold disappears northward away from Karakorum Fault. (Photo by E. Wang)


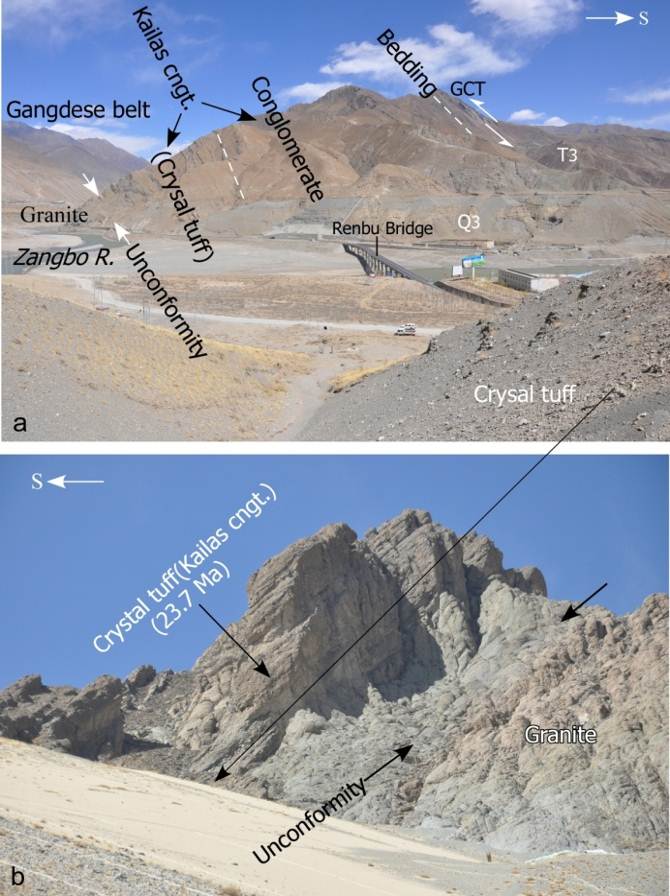


**SI*1-7*** a: View to the west along the southern edge of the middle part of the Gangdese magmatic belt, in an area 10 km north of Renbu. The Kailas unit here dips to the south, unconformably overlies granite, and is overthrust from the south by Triassic (T) flysch upon the GCT. The Kailas unit consists of two parts: a lower crystal tuff and overlying conglomerate; b: Close-up view of the unconformity between the crystal tuff unit and granite. Zircon crystals from the tuff have 22.34±0.22 Ma age (see **SI*2-2*** for details). (Photo by E. Wang)


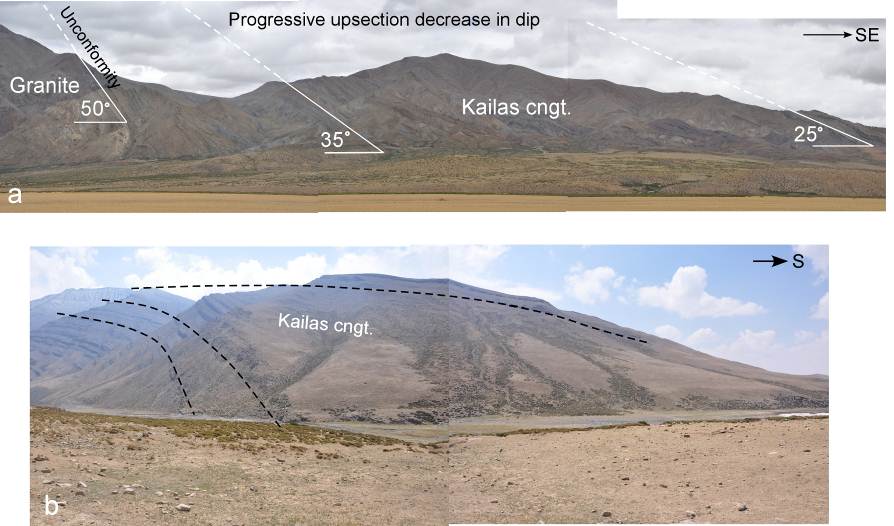


**SI*1-8***a: View northeast of the lower part of the Kailas unit unconformably overlying granite along the southern edge of the Gangdese belt in an area 15 km west of Mt. Kailas, where the sequence decreases in dip upsection to the southeast; b: View east of Kailas conglomerate with bedding curving over to the south along the southern edge of the western part of the Gangdese belt, also showing a progressive decrease in dip upsection in the area 5 km west of Mt. Kailas. (Photo by E. Wang)


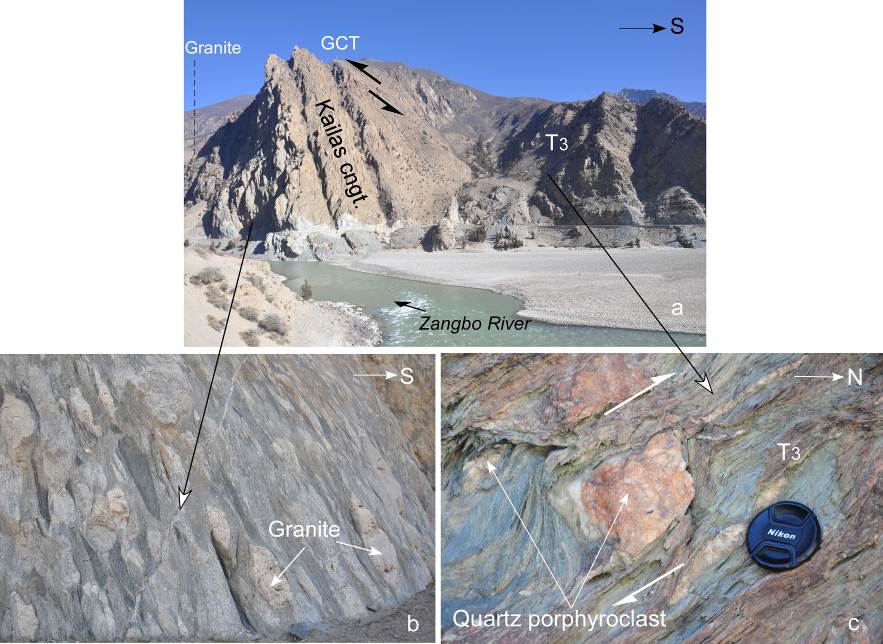


**SI*1-9***a, b: Outcrop view of components of the Gangdese belt in the Langxian area showing ductile shear deformation within the GCT. We have recorded stretch lineations within the mylonite striking NE-SW, oblique to the strike of the foliation, indicating that the top-to-north movement along the GCT had a left-lateral slip component. Note that all sedimentary clasts are composed of granite; c: An outcrop view of Late Triassic Tethyan flysch of the hanging wall of the GCT with S-C fabric and rotated quartz porphyroclasts indicating top-to-north displacement on the GCT. (Photo by E. Wang)


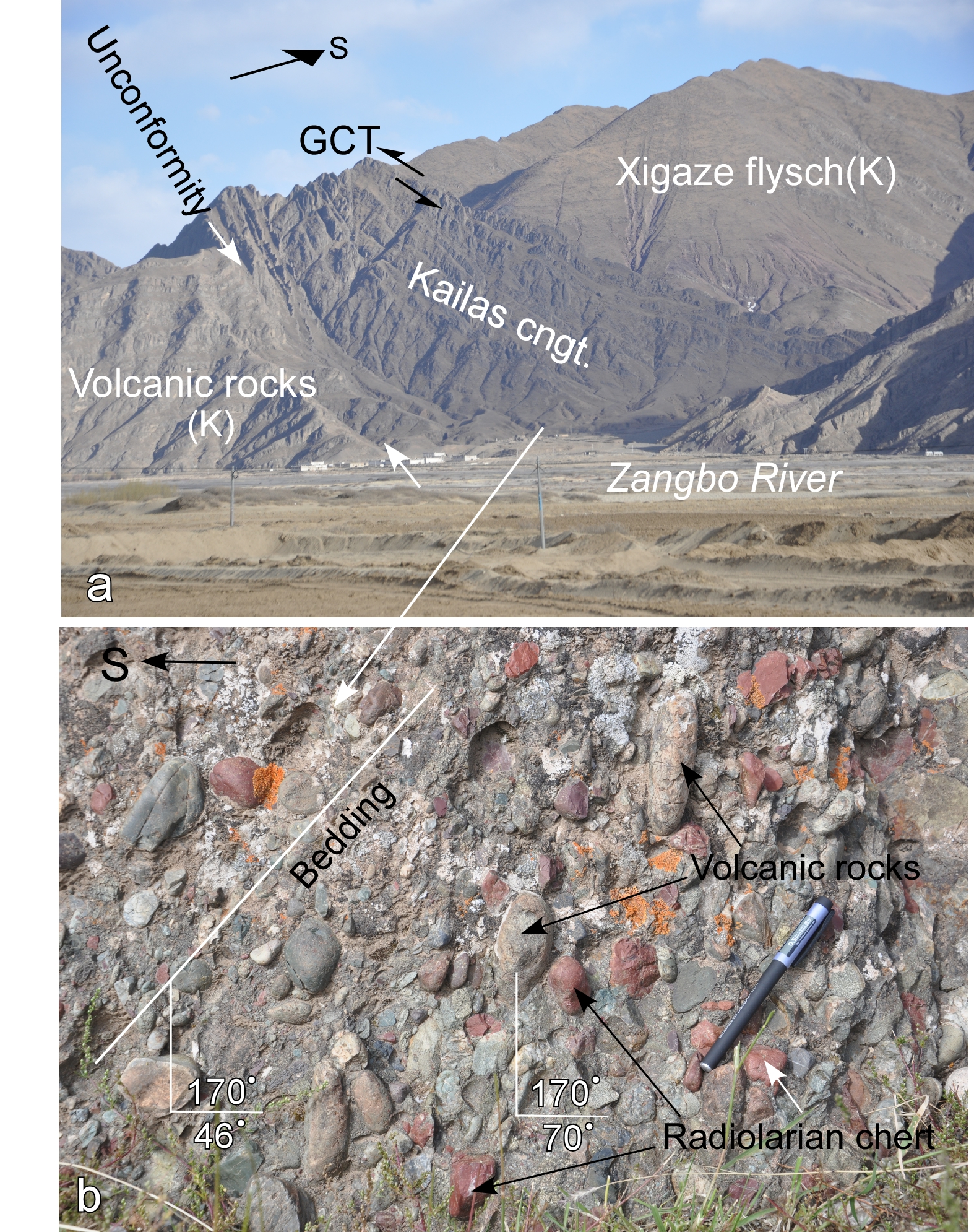


**SI*1-10*** a: View to the southeast of Kailas conglomerate 35 km northwest of Xigaze, showing a fold overturned to the north in the footwall of the GCT; b: The Kailas conglomerate in this section contains numerous clasts identical in lithology and age to the ophiolite suite within the Zangbo suture zone complex—purple colored chert and mafic and ultra-mafic clasts (See SI*2-3)*. Note that many flat pebbles made up of the radiolarian chert consistently dip to the south, indicating that these clasts were transported from the Zangbo suture zone to the south. See **SI*1-13*** for location. (Photo by E. Wang)


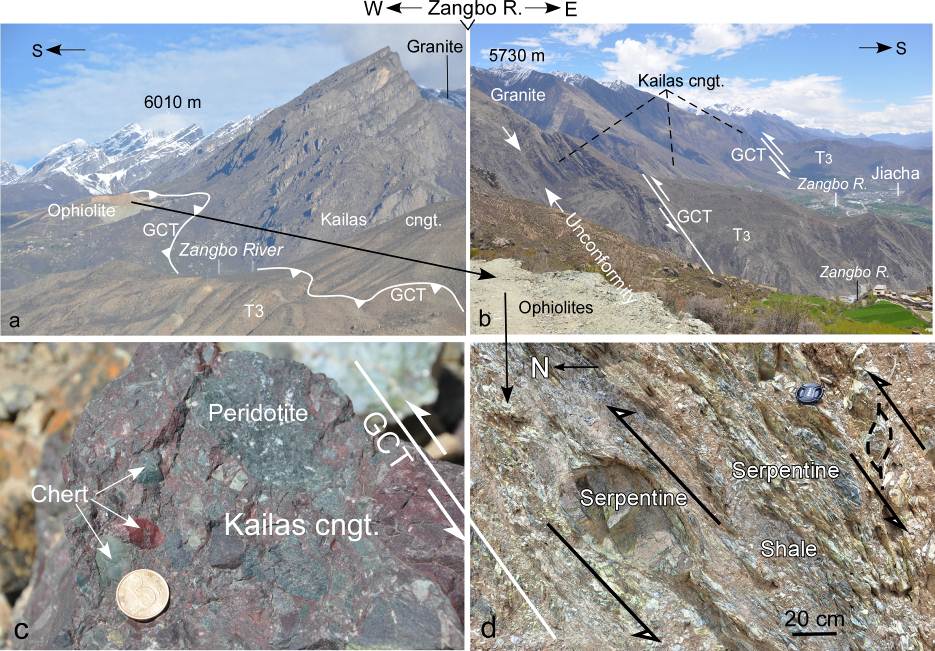


**SI*1-11***a-b: View of the steep southern edge of the Gangdese belt, divided into two parts by the Zangbo River in the Jiacha area, defined by the Kailas conglomerate unit dipping to the south at 45°. The Kailas conglomerate is overlain to the south by ophiolitic mélange of the Zangbo suture zone and Late Triassic (T_3_) flysch of the Tethyan Himalaya, both transported on the GCT, which itself dips to the south at 50° (see **SI*1-1*** and ***1-16*** for location); c: Close-up view of clasts within Kailas conglomerate in the footwall of the GCT in a-b. The clasts are mainly composed of ultra-mafic and mafic rocks, purple-colored chert and volcanic rocks, which also occur within the hanging wall of the GCT; d: Close-up view of the GCT in the section shown in b (above), separating ophiolite mélange of the hanging wall from Kailas conglomerate in the footwall. Peridotite clasts in the mélange are considered to have been serpentinized during shearing along the GCT and have been incorporated with Triassic phylitte material, both showing S-C fabric indicating displacement of the hanging to the south. (Photo by E. Wang)


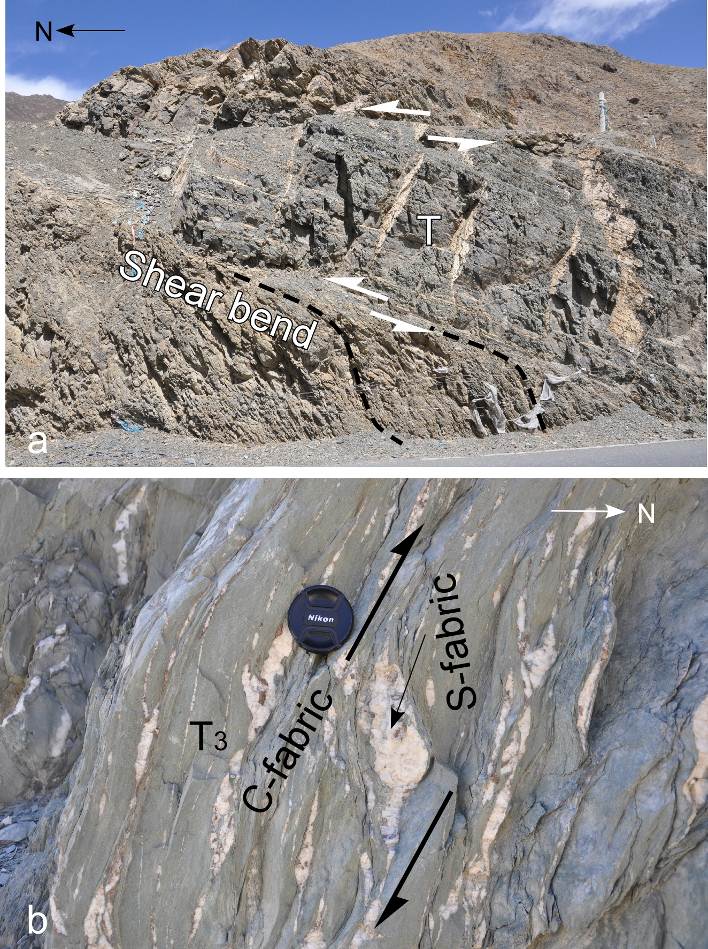


**SI*1-12*** a: Close-up of Late Triassic Tethyan sequence flysch (T_3_) within the GCT, exhibiting a shear bend indicating top-to-north movement along the GCT, in the Renbu area; b: Close-up of Late Triassic Tethyan sequence flysch within the GCT showing typical S-C shear fabric demonstrating top-to-north displacement in the Langxian area. (Photo by E. Wang)

**
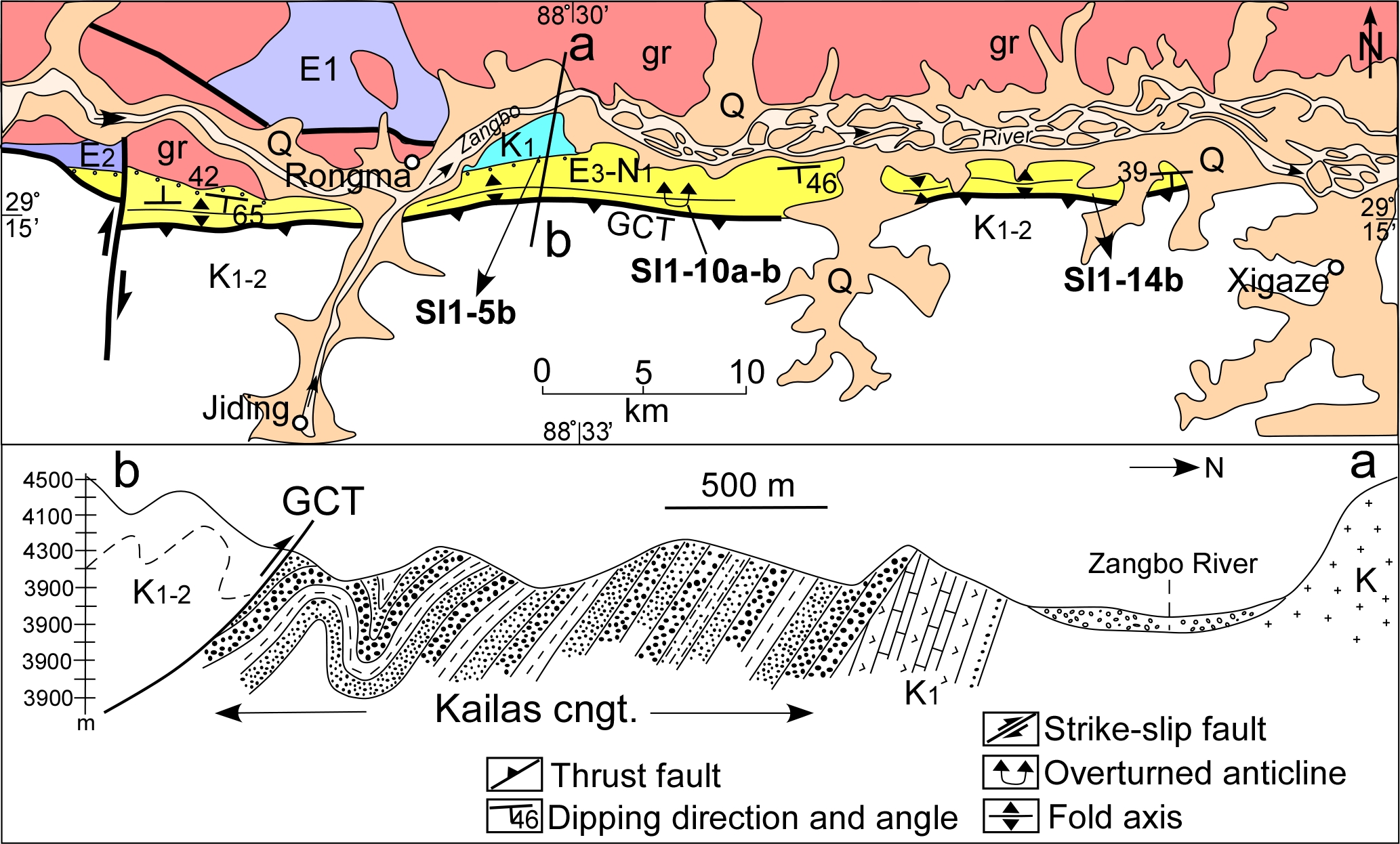
**

**SI*1-13*** Geological map of the Xigaze area showing Kailas conglomerate overlain by Xigaze flysch (K_1-2_) across the GCT. The Kailas conglomerate in this area contains a large number of mafic, ultra-mafic and the multi-colored radiolarian chert clasts. Locations of **SI*1-5b***, ***-10a-b***, ***-14b*** are shown; below: Cross-section a-b in map of the southern margin of the Gangdese magmatic belt. Q: Quaternary; K_1-2_: Cretaceous (Xigaze flysch); E_2_: Late Eocene (Qiuwu conglomerate); K_1_: Early Cretaceous; gr: Granite.


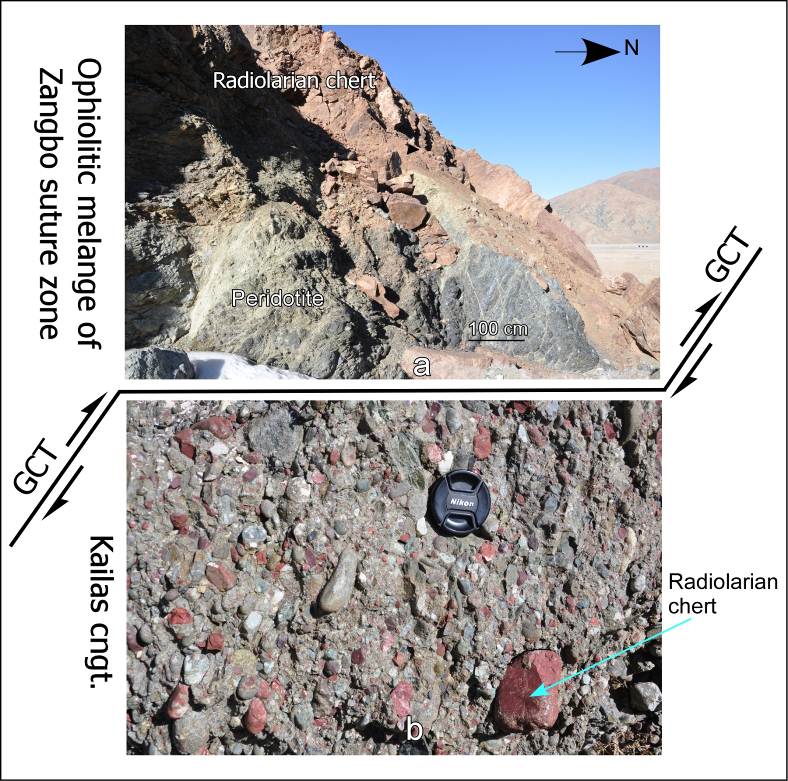


**SI*1-14*** Composited figure of outcrop sections either side of the GCT in the vicinity of Xigaze, showing radiolarian chert of similar age range, amongst other lithologies. In the upper photo (a), radiolarian fossils with Middle Jurassic ages have been identified (see **SI*3-1***) from chert within the ophiolite suite of the Zangbo suture zone forming the hanging wall of the GCT in the Bailang area, 10 km south of Xigaze, although this unit is generally dated on maps as Late Jurassic-Cretaceous. In photo (b), Late Jurassic to Cretaceous radiolarian fossils occur (see **SI*3-1***) within the purple-colored chert clasts evident in the Kailas conglomerate occurring in the footwall of the GCT at this site 10 km northwest of Xigaze. See **SI*1-13*** for the location. (Photo by E. Wang)


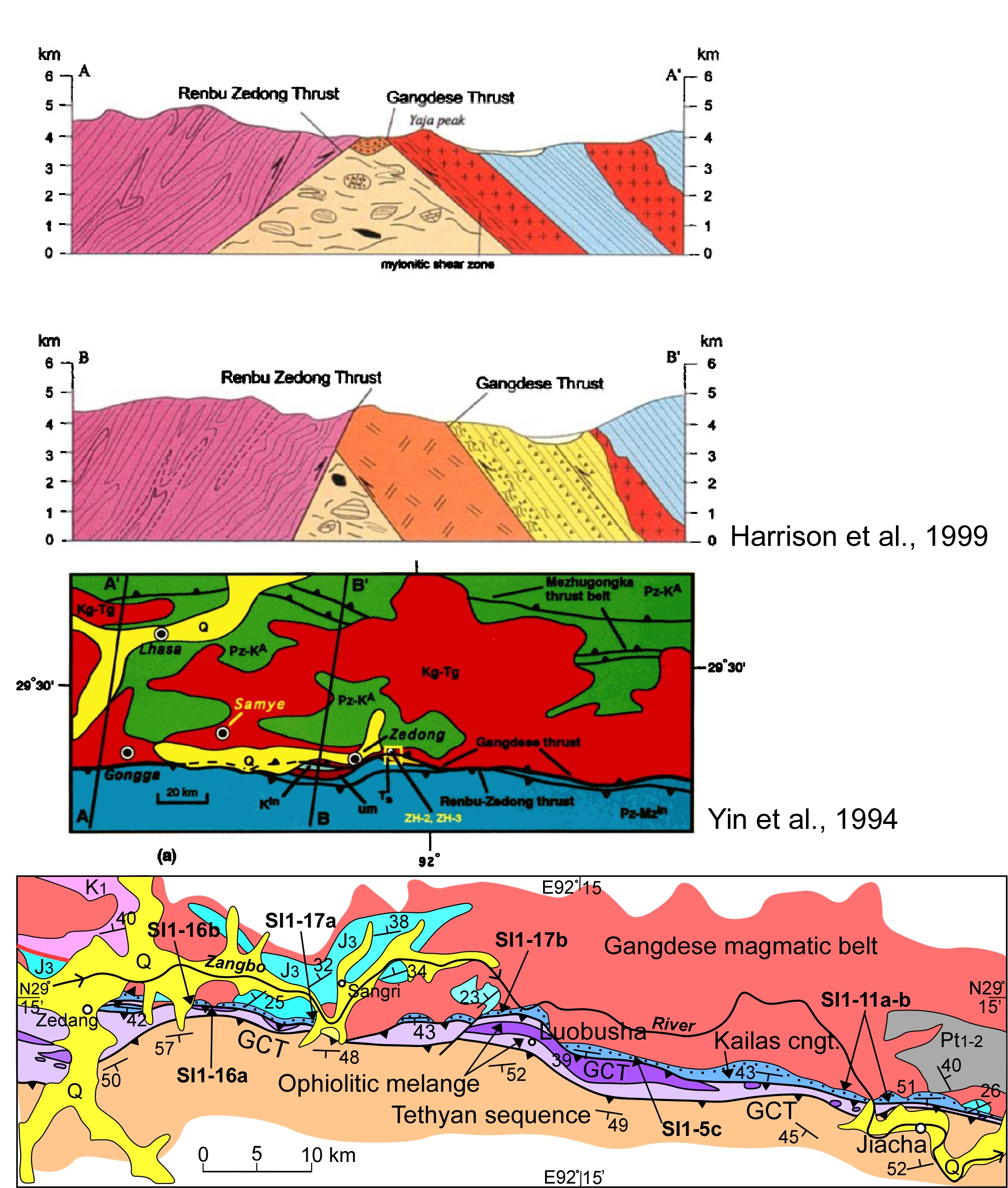


**SI*1-15*** Upper: Structural cross-section of the Zedang window made by Mark Harrison et al. (1999), showing that the Kailas cngt. is covered by the ophiolitic mélange along the GCT on the south and by the Zedang structural window (Yajia peak) along the Gangdese Thrust on the north; Middle: Tectonic map of the Zedang area, made by An Yin, et al. (1994), showing that the Kailas conglomerate is cut by two thrust faults, the Renbu-Zedang fault (GCT) on the south and the Gangdese Thrust on the north; Lower: Geological map of the Zedang area made in this study, showing that the Kailas conglomerate is only cut by the GCT on the south, with its northern boundary marked by the unconformity, dividing the Gangdese batholiths to the north. Field observations of the contact relationships between Kailas conglomerate and other rocks are shown in **SI*1-16~17*.**


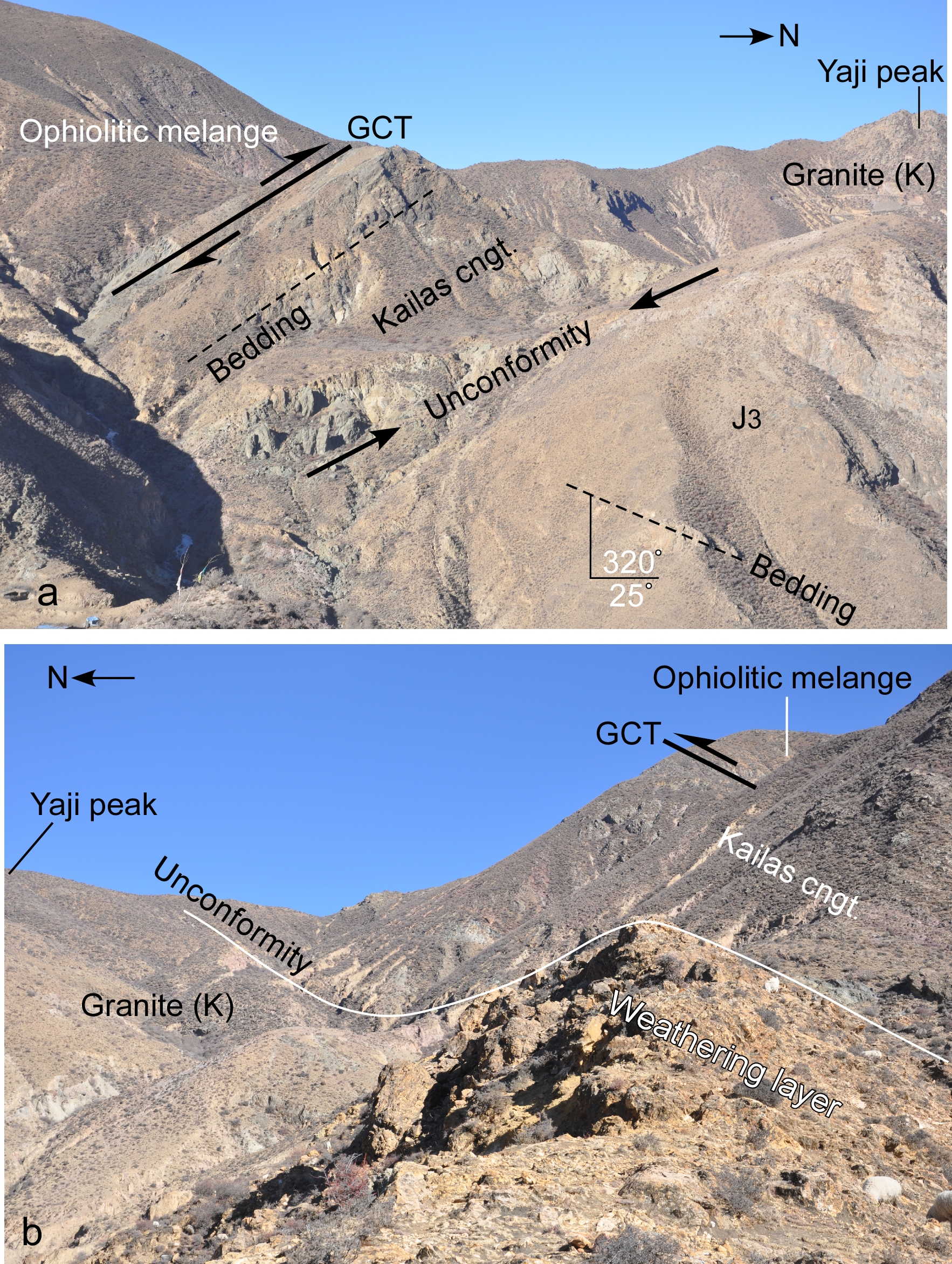


**SI*1-16***a: View to the west of Kailas conglomerate, 10 km west of Zedang. The Kailas unit here dips south and is covered by Ophiolitic melange along the GCT and unconformably overly Cretaceous granite (Yajia peak) and its country rocks of Late Jurassic volcanic rocks to the north (J3). Note that there is no Gangdese Thrust which placed the granite and Late Jurassic rocks as a part of structural widow over the conglomerates, as illustrated in Refs: 1, 4; b: View to the east of the unconformity between Kailas conglomerate and Gangdese belt granite (Yajia peak), both in the footwall of the GCT, 2000 m west of the section shown in photo a, above. Note that the unconformity is marked by a weathering layer of granite (foreground) and there is no intervening Gangdese Thrust. (Photo by E. Wang)


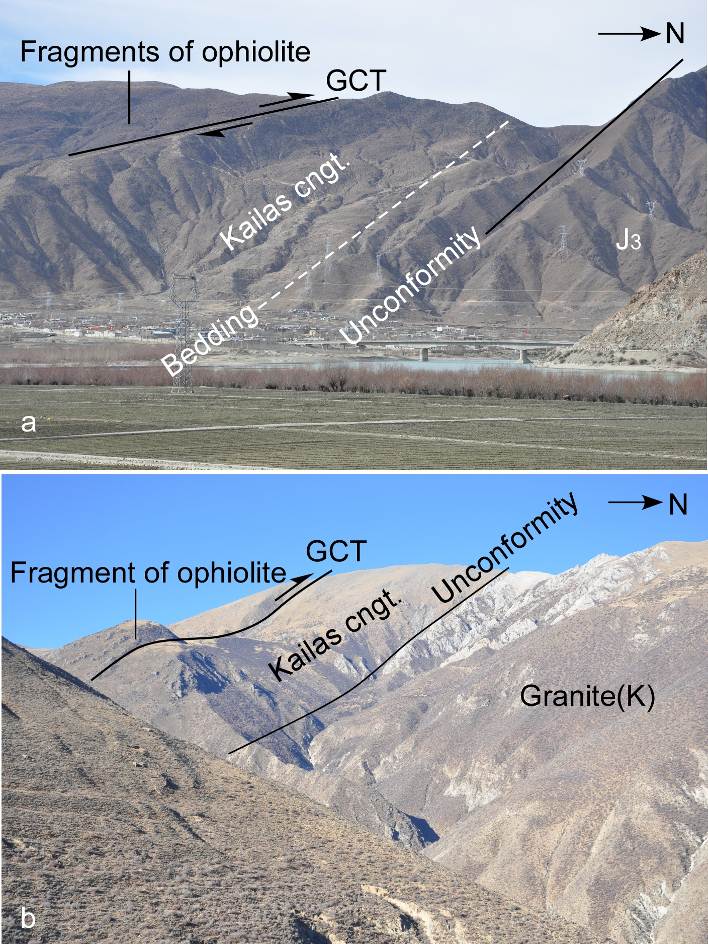


**SI*1-17*** a: View to the west of the southern edge of the Gangdese belt in the area 10 km east of Zedang. The Kailas conglomerate here dips to the south, overlying Late Jurassic volcanic rocks (J_3_), and to the south is structurally overlain by fragments of ophiolite along the GCT. Note that the Late Jurassic (J_3_) here was defined to be part of a structural window and considered (Refs 1,4), incorrectly in our view, to structurally overlie the Kailas conglomerate along a Gangdese Thrust; b: View to the west at the southern edge of the Gangdese belt in the area of Luobusha, showing that the Kailas conglomerate is only cut by the GCT, there being no evidence for the so-called the Gangdese Thrust, as defined in prior work (Refs 1, 4). (Photo by E. Wang)

**
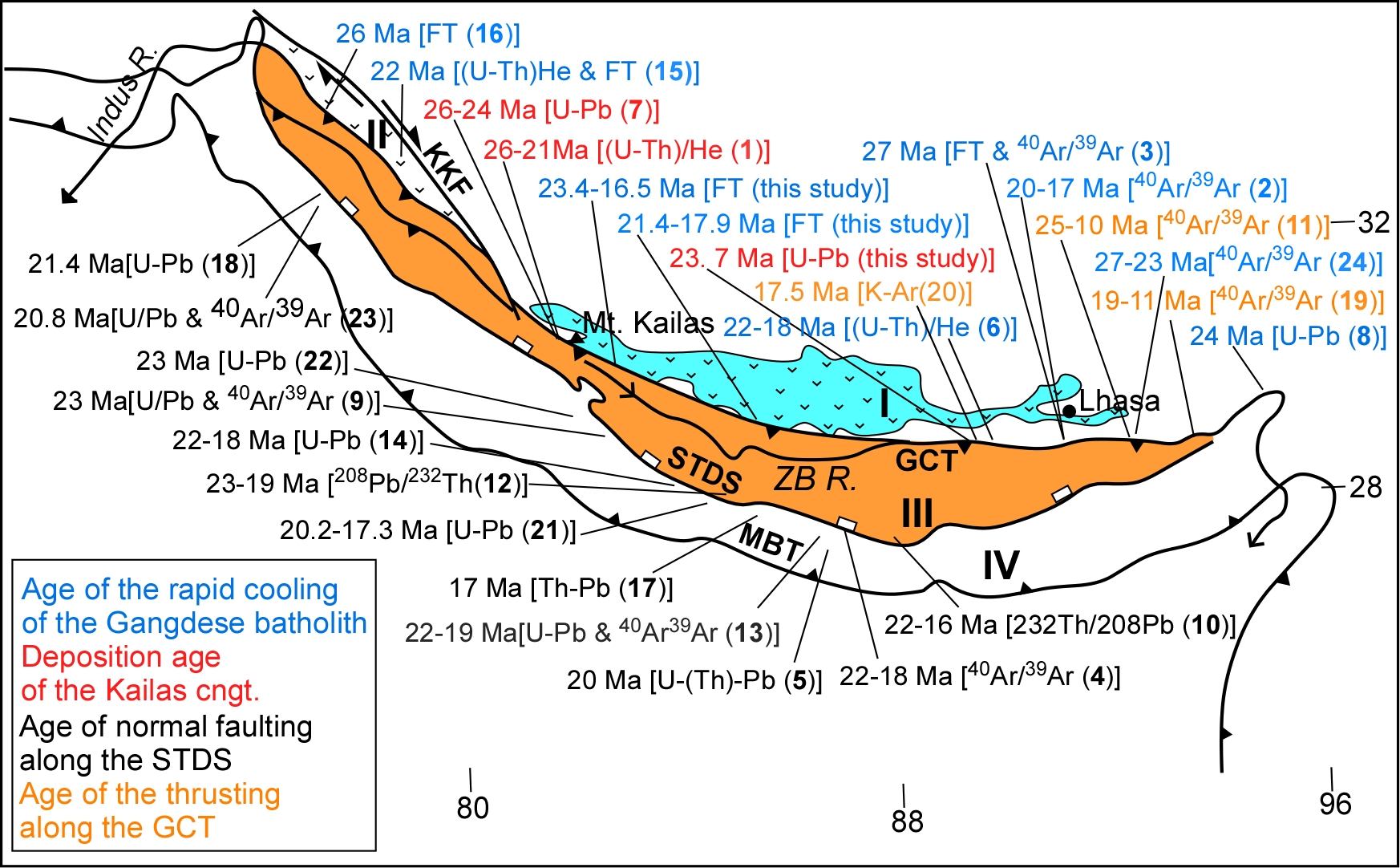
**

**SI*1-18*** Tectonic sketch map of the southern margin of the Tibetan plateau showing age estimates for normal fault offset on the South Tibet Detachment System (STDS) and for cooling/exhumation of the Gangdese magmatic belt. KKF = Karakorum fault; I = Gangdese magmatic belt; II = Ladakh belt; III = Tethyan belt; IV = High and Lesser Himalaya

***References***

1. Carrapa, B, Orme DA, DeCelles PG, Kapp P, Cosca MA, Waldrip R (2014) Miocene burial and exhumation of the India-Asia collision zone in southern Tibet: Response to slab dynamics and erosion. *Geology* 42: 443–446, doi:10.1130/G35350.1.
2. Copeland P, Harrison MT, Kidd WSF (1987) Rapid early Miocene acceleration of uplift in the Gangdese Belt, Xizang (southern Tibet), and its bearing on accommodation mechanisms of the India-Asia collision. *Earth Planet. Sci. Lett*.86:240–252.
3. Copeland P, Harrison TM, Pan Y, Kidd WSF, Roden M, Zhang Y Q (1995) Thermal evolution of the Gangdese Batholith, southern Tibet: a history of episodic unroofing. *Tectonics* 14:223–236.
4. Coleman ME, Hodges KV (1998) Contrasting Oligocene and Miocene thermal histories from the hanging wall and footwall of the South Tibetan detachment in the central Himalaya from 40Ar/39Ar thermochronology, Marsyandi valley, central Nepal, *Tectonics*, 17 (5): 726-740.
5. Cottle J, Micah M, Jessup J, Dennis LN, Searle MP, Richard DL, Matthew S, Horstwood A (2007) Structural insights into the early stages of exhumation along an orogen-scale detachment: The South Tibetan Detachment System, Dzakaa Chu section, Eastern Himalaya, *Journal of Structural Geology* 3: 1-17.
6. Dai J, Wang C, Hourigan J, Li Z, Zhuang G (2013) Exhumation History of the Gangdese Batholith, Southern Tibetan Plateau: Evidence from Apatite and Zircon (U-Th)/He Thermochronology, The Journal of Geology 121: 155-172. DOI: 10.1086/669250.
7. DeCelles PG, Kapp P, Quade J, Gehrel, GE (2011)Oligocene–Miocene Kailas basin, southwestern Tibet: Record of postcollisional upper-plate extension in the Indus-Yarlung suture zone，*GSA Bulletin*，123: 1337–1362，doi: 10.1130/B30258.1.
8. Ding L, Zhong DL, Yin A, Kapp P, Harrison TM (2001) Cenozoic structural and metamorphic evolution of the eastern Himalayan syntaxis (Namche Barwa). Earth and Planetary Science Letters 192: 423–438.
9. Godin L, Parrish RR, Brown R, Hodges KV (2001) Crustal thickening leading to exhumation of the Himalayan metamorphic core of central Nepal: Insight from U-Pb geochronology and 40Ar/39Ar thermochrology. *Tectonics* 20: 729-747.
10. Grujic D, Hollister LS, Parrish RR (2002) Himalayan metamorphic sequence as an orogenic channel: Insight from Bhutan, *Earth Planet. Sci Lett*. 198: 177-191, doi: 10.1016/S0012-821X(02)00482-X.
11. Harrison TM, Yin A, Grove M, Lovera OM (2000) The Zedang window: A record of superposed Tertiary convergence in southeastern Tibet, *Journal of Geophysics Research* 105: 19,211-19,230.
12. Harrison TM, Grove M, Mckeegan KD., Coath CD, Lovera OM, Lefort P (1999) Origin and episodic emplacement of the Manaslu Intrusive Complex, Central Himalaya. *Journal of Petrology* 40: 3–19.
13. Hodges KV, Parrish RR, Housh TB, Lux DR, Burchfiel BC, Royden LH, Chen Z (1992) Simultaneous Miocene extension and shortening in the Himalayan orogen. *Science* 258: 1466–1470.
14. Hodges KV, Parrish RR, Searle MP (1996) Tectonic evolution of the central Annapurna Range, Nepalese Himalayas. *Tectonics* 15:1264–91.
15. Kirstein LA, Sinclair H, Stuart FM, Dobson K (2006) Rapid early Miocene exhumation of the Ladakh Batholith, western Himalaya. *Geology* 34:1049–1052.
16. Kirstein LA (2011) Thermal evolution and exhumation of the Ladakh Batholith, northwest Himalaya, India. *Tectonophysics* 503: 222–233.
17. Murphy MA, Harrison TM (1999) Relationship between leucogranites and the Qomolangma detachment in the Rongbuk Valley, south Tibet. *Geology* 27: 831 – 834.
18. Noble SR, Searle MP (1995) Age of crustal melting and leucogranite formation from U-Pb zircon and monazite dating in the western Himalaya, Zanskar, India. *Geology* 23: 1135–1137.
19. Quidelleur X, Grove M, Lovera OM, Harrison TM, Yin A (1997) Thermal evolution and slip history of the Renbu-Zedang Thrust, southeastern Tibet. *J. Geophys. Res.* 102: 2659-2679.
20. Ratschbacher L, Frisch W, Liu G (1994) Distributed deformation in southern and western Tibet during and after the India-Asia collision. *J Geophys Res* 99: 19,917-19,914.
21. Searle MP, Parrish RR, Hodges KV, Hurford AJ, Ayres MW, Whitehouse, MJ (1997) Shisha Pangma leucogranite, south Tibetan Himalaya: field relations, geochemistry, age, origin and emplacement. *Journal of Geology* 105:295–317.
22. Searle MP, Noble SR, Hurford AJ, Rex DC (1999) Age of crustal melting, emplacement and exhumation history of the Shivling leucogranite, Garhwal Himalaya, *Geol. Mag.* 136: 513-525.
23. Walker JD, Martin MW, Bowring SA, Searle MP, Waters DJ, Hodges KV (1999) Metamorphism, melting, and extension: age constraints from the High Himalayan slab of southeast Zanskar and northwest Lahaul. *Journal of Geology* 107: 473 – 495.
24. Yin A, Harrison TM, Ryerson FJ, Chen W, Kidd WSF, Copeland P (1994) Tertiary structural evolution of the Gangdese Thrust system, southeastern Tibet. *J. Geophys Res.* 99:18,175–18,201.

**
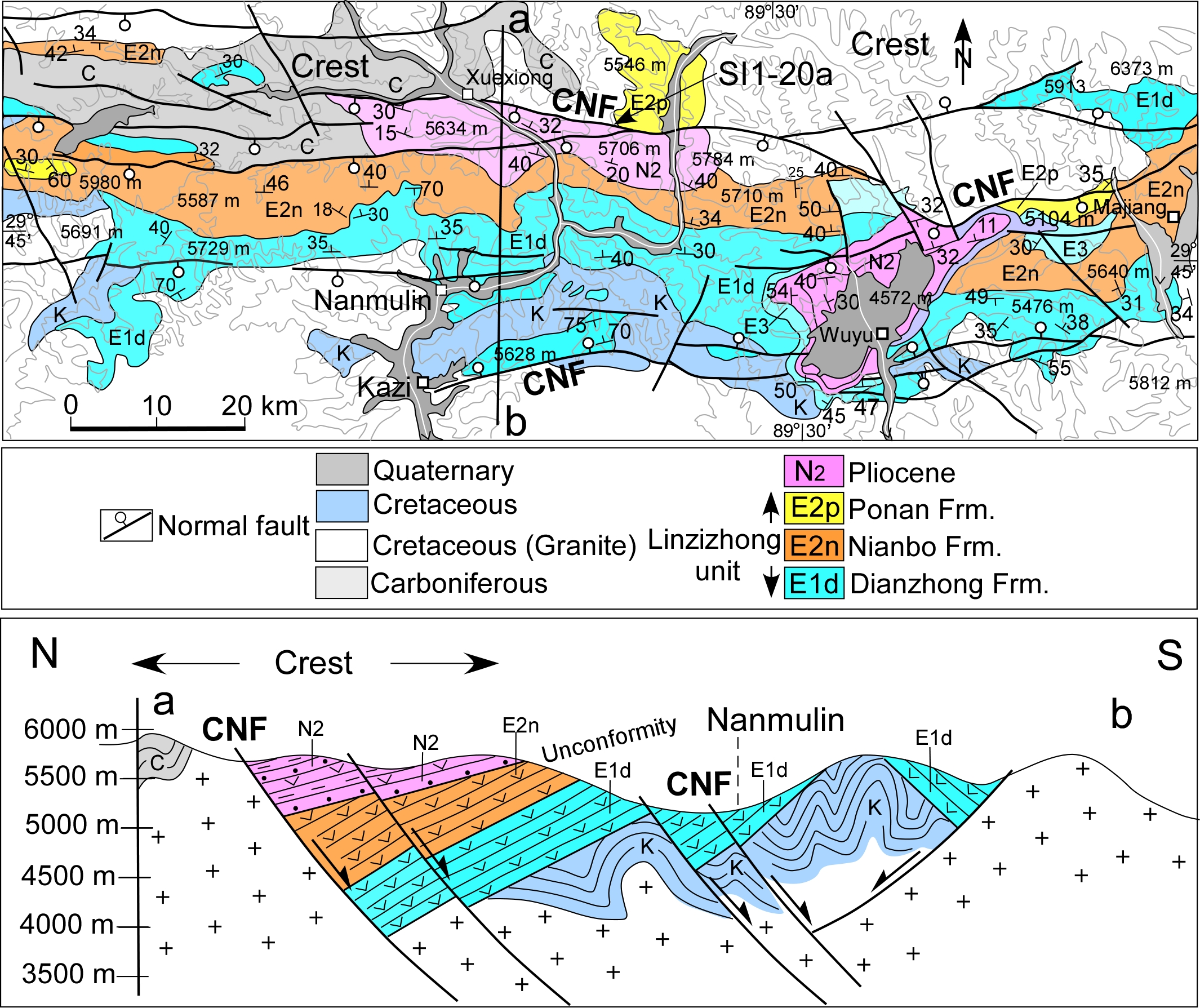
**

**SI*1-19*** Geological map and north-south cross-section of Nanmulin area, showing normal offset on the Crest Normal Fault Zone (CNF) and related faults.

**
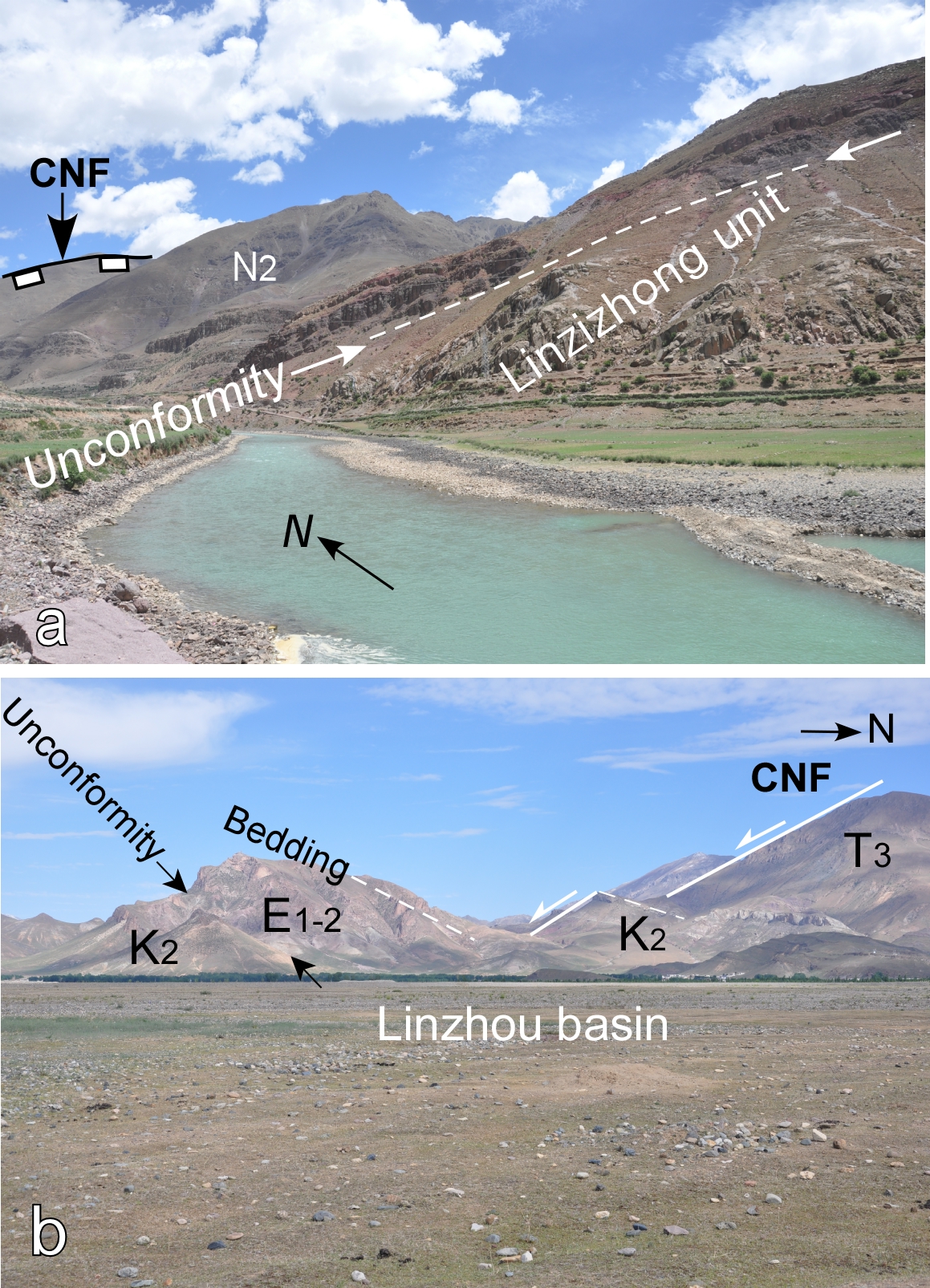
**

**SI*1-20*** a: View north at the crest of the middle part of the Gangdese belt in the area north of Nanmulin, underlain by both the Linzizhong volcanic rocks separated by a shallow dipping unconformity surface from purple-colored clastic and volcanic rocks of Pliocene age, all of which gently tilt to the north along a south-dipping normal fault, namely the Crest Normal Fault Zone (CNFZ). The tilting probably started after deposition of the Linzizhong unit and persisted to the Pliocene. (Photo by E. Wang)


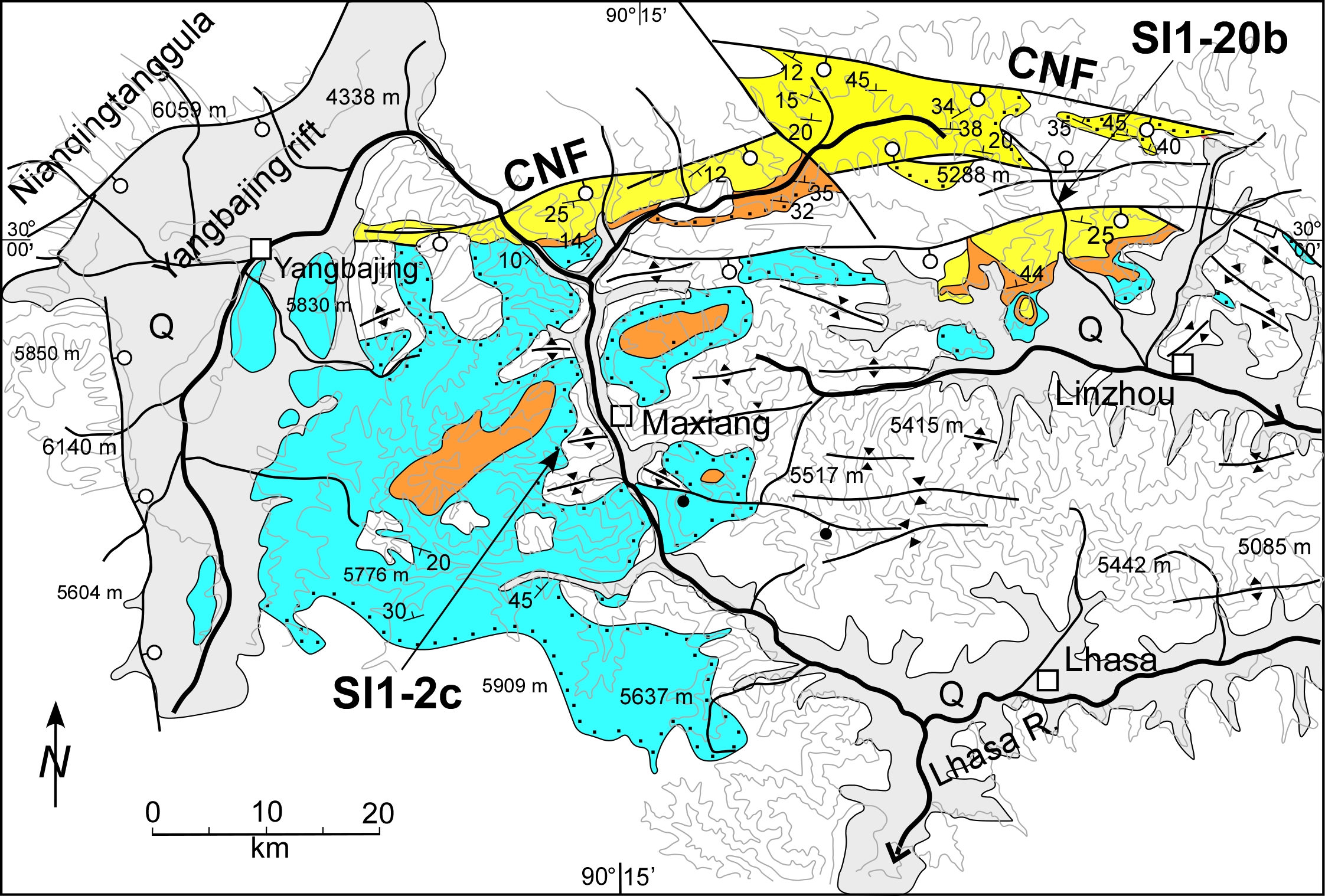


**SI*1-21*** Geological map of the Lhasa area, showing normal offset on the Crest Normal Fault Zone (CNF) and related faults. Map symbols are the same as those used in **SI*1-19***.

**SI*2-1* Results of fission track analysis**

Here we report zircon and apatite fission track thermochronology ages for granite samples collected from two transects across the Gangdese belt. Seven samples were collected from the Napijia (NPJ) section at a longitude of 82° (Figure SI2-1-1) E and at elevations ranging from 5100 to 5750 m a.s.l., and three samples were collected from section Ajuexiong (AJX) (Figure SI2-1-2) at longitude 86°E and at elevations 5150-5500 m a.s.l. All samples yielded good quality zircon and apatite crystals that could easily be analysed.

***Fission track results***

A zircon fission track age of 56.2±4.2 Ma was derived from the more western transect (sample GDS 12-1, Table SI2-1-1). As this sample derives from a magmatic rock unit it is difficult to infer whether this age reflects magmatic cooling following intrusion of the granitoid to ambient temperatures in the crust, or whether it also includes a component of post-emplacement cooling by exhumation of the granitoid through 280°C, the closure temperature for fission tracks in zircon, or a combination of both. The important observation here is that the zircon fission track age for this sample lies within the 110-45 Ma interval over which magmatism occurred within the Gangdese belt and therefore magmatic cooling is probably the main control on the age. Sample AJX 12-1 was derived from the more centrally located transect AJX and has a zircon fission track age of 50.9±2.6 Ma. We interpret these data in the same way: mainly magmatic cooling during the final stages of Gangdese belt magmatism.

We report seven apatite fission track ages for the NPJ section. They have mean ages ranging between 16.5 and 23.4 Ma (Table SI2-1-2). Less than the optimum number of horizontally confined track lengths could be measured, but those that were measured revealed long mean track lengths mostly in the range 14.5 to 15.5 microns (Table Sl2-1-2). This indicates that the observed ages arise from rapid Early Miocene cooling from ambient temperatures higher than the closure temperature of around 110 °C. The observed ages do not show a systematic change with elevation, which emphasises that they represent one group from a crustal section that lay below the pre-cooling partial annealing zone, and a section that cooled rapidly via exhumation to ambient temperatures less than 60°C during the Early Miocene.

The apatite fission track ages from the AJX Section lie in the range 17.9 to 21.4 Ma and have mean track lengths between 12.4 and 15.4 microns, with less than the optimal number of track lengths measured. We consider these samples to have the same cooling history considering their proximity to each other (only 350 m elevation difference) and their similar Early Miocene ages. We interpret the geological signal to be essentially the same as that for the NPJ section: a rock section that was rapidly exhumed from temperatures higher than 110°C to less than 60°C during the Early Miocene. It is likely that exhumation continued into the Recent, albeit that after the early Miocene the rates were more gradual.

***Fission track methods***

Sample preparation and experimental methods used in this study follow those reported by Green (1985) and Gleadow et al. (1986), as adopted in the University of Waikato Fission Track laboratory (Kamp et al. 1989; Kamp et al. 1992). Apatite and zircon concentrates were separated from 2 kg samples of basement rocks using standard magnetic and heavy liquid techniques. The external detector method (Gleadow et al. 1981) has been used exclusively in this study. Teflon zircon mounts were etched in NaOH:KOH eutectic solution at 230±1°C between 10 and 18 hours. Apatite and zircon mounts were irradiated in the ANSTO reactor at Lucas Heights, Sydney, Australia, with nominal fluences of 1×10^16^ n/cm^2^ for apatite and 2×10^15^ cm^2^ for zircon. The fission track ages were determined using the zeta calibration method (Green, 1985; Hurford and Green, 1982) and calculated as central ages (Galbraith and Green 1990). Confined track lengths in apatite were measured using a digitizing tablet connected to a computer, superimposed on the microscope field of view via a projection tube. This system was calibrated against a stage graticule ruled in 2 micron divisions. Tracks with this system can be measured with a precision of 0.2 microns. Tracks were measured using the recommendations of Laslett et al. (1982).

***References***

1. Gleadow, A.J.W. 1981: Fission track dating methods: what are the real alternatives? Nuclear Tracks Radiation Measurements 5, 15-25.
2. Gleadow, A.J. W., Duddy, I.R., Green, P.F. and Hegarty, K.A. 1986: Fission track lengths in the apatite annealing zone and the interpretation of mixed ages. Earth and Planetary Science Letters 78, 245-254.
3. Green, P.F. 1985: Comparison of zeta calibration baseline for fission track dating of apatite, zircon and sphere. Chemical Geology 58, 1-22.
4. Hurford, A.J. and Green, P.F. 1982: A guide to fission track dating calibration. Earth Science Letters 59, 343-354.
5. Kamp, P.J.J., Green P.F. and White S.H. 1989: Fission track analysis reveals character of collisional tectonics in New Zealand. Tectonics 8, 169-195.
6. Kamp, P.J.J., Green P.F. and Tippett J.M. 1992: Tectonic architecture of the mountain front-foreland basin transition, South Island, New Zealand, assessed by fission track analysis. Tectonics 11, 98-113.
7. Laslett, G.M., Kendall, W.S., Gleadow, A.J.W. and Duddy, I.R. 1982: Bias in the measurement of fission track length distributions. Nuclear Tracks Radiation Measurements 6, 79-85.

**
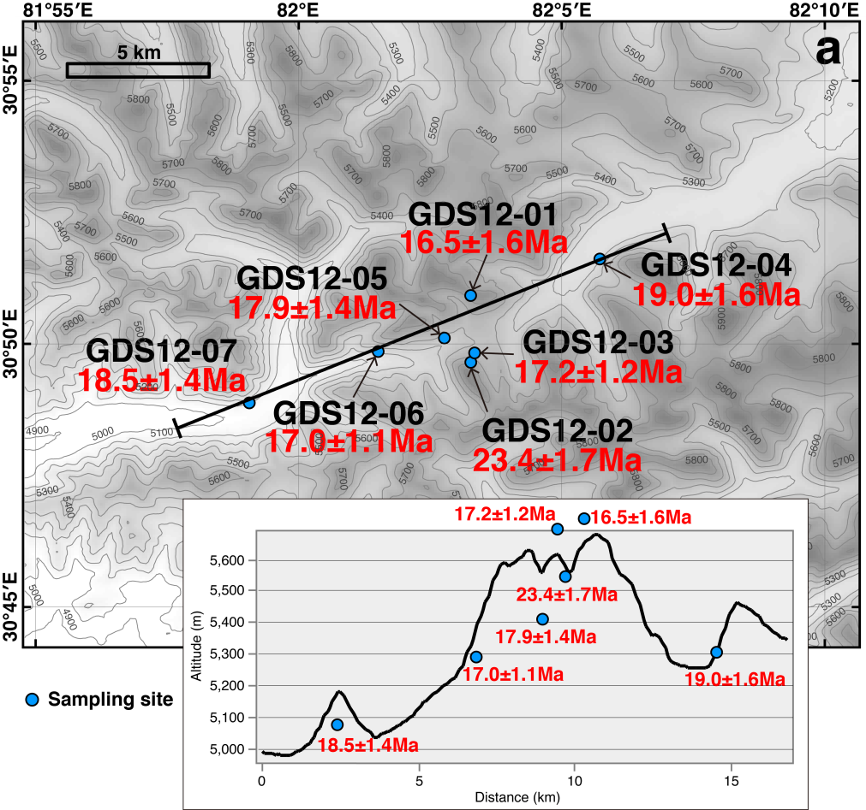
**

Figure SI2-1-1 Napijia (NPJ) section

**
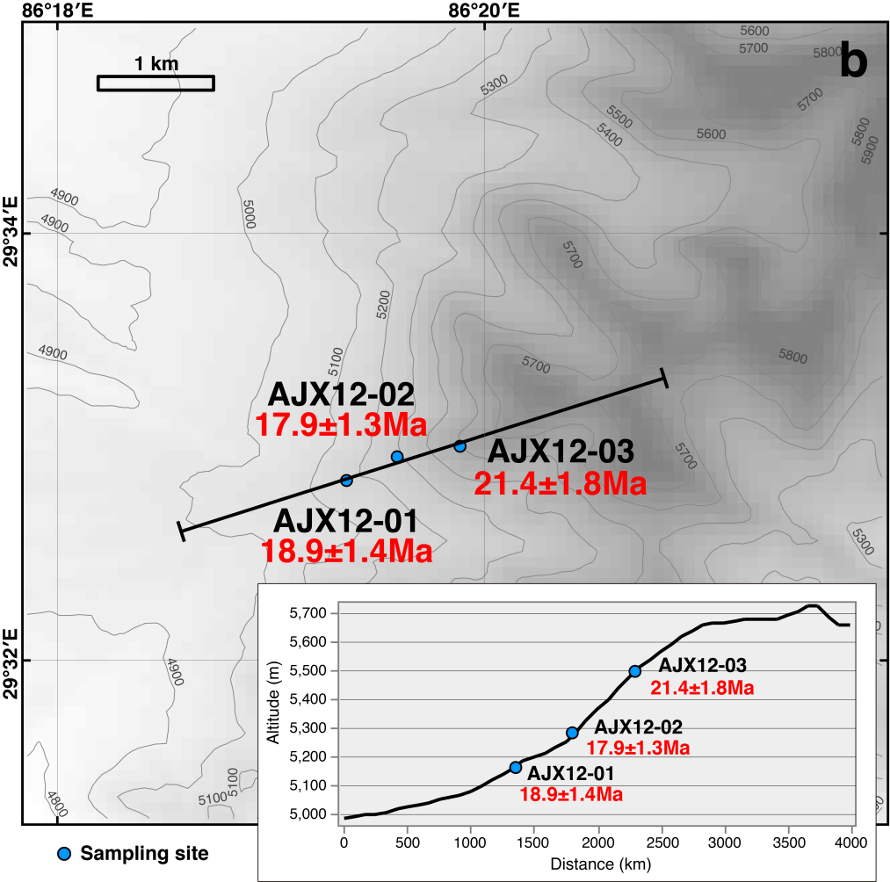
**

Figure SI2-1-2 Ajuexiong (AJX) section

Table SI2-1-1: Gangdese Fission Track Zircon Age.

| Sample No. | Number  of crystals | Spontaneous | | Induced | | P(χ^2^) % | ρd | Nd | Age (Ma)  ±1σ |
| --- | --- | --- | --- | --- | --- | --- | --- | --- | --- |
|  |  | ρs | Ns | ρi | Ni |  |  |  |  |
| *AJX section* | | | | | | | | | |
| AJX12-1 | 15 | 15.48 | 2733 | 22.08 | 3898 | 0 | 1.095 | 2579 | 50.9±2.6 |
| *NPJ section* | | | | | | | | | |
| GDS12-1 | 15 | 20.25 | 1941 | 26.02 | 2494 | 0 | 1.100 | 2610 | 56.2±4.2 |

Track densities (ρ) are ×10^6^ tracks cm^-2^. All analyses are by the External Detector Method using 0.5 for the 4π/2π geometry correction factor. Zircon ages calculated using dosimeter glass CN1 and zeta-CN1=133.5±1.1 (±1σ). P(χ^2^) is the probability of obtaining χ^2^ value for v degrees of freedom (where v is the number of crystals -1)(Galbraith, 1981); pooled ρs/ρi ratio is used to calculate age and uncertainty where P(χ^2^) >5%; mean ρs/ρi ratio is reported for samples where P(χ^2^) <5% and for which Central ages (Galbraith and Green, 1990) are calculated.

Table SI2-1-2: Gangdese Fission Track Apatite Age and Track Length

| Sample No. | Number  of crystals | Spontaneous | | Induced | | P(χ^2^) % | ρd | Nd | Age (Ma)  ±1σ | Mean Track Length (μm)  ±1σ | Standard Deviation (μm) | Number of Length |
| --- | --- | --- | --- | --- | --- | --- | --- | --- | --- | --- | --- | --- |
|  |  | ρs | Ns | ρi | Ni |  |  |  |  |  |  |  |
| *AJX section* | | | | | | | | | | | | |
| AJX12-1 | 20 | 0.262 | 212 | 2.946 | 2385 | 99.2 | 1.221 | 2897 | 18.9±1.4 | 14.75± 0.28 | 1.21 | 19 |
| AJX12-2 | 20 | 0.342 | 240 | 4.055 | 2844 | 98.7 | 1.216 | 2885 | 17.9±1.3 | 12.40± 1.32 | 3.95 | 9 |
| AJX12-3 | 20 | 0.191 | 166 | 1.814 | 1577 | 89.4 | 1.169 | 2774 | 21.4±1.8 | 15.39±1.14 | 1.97 | 3 |
| *NPJ section* | | | | | | | | | | | | |
| GDS12-1 | 20 | 0.209 | 131 | 2.666 | 1670 | 99.9 | 1.211 | 2873 | 16.5±1.6 | 15.52±0.43 | 1.59 | 14 |
| GDS12-2 | 21 | 0.267 | 225 | 2.378 | 2006 | 65.5 | 1.200 | 2848 | 23.4±1.7 |  |  |  |
| GDS12-3 | 20 | 0.372 | 237 | 4.491 | 2864 | 61.5 | 1.195 | 2836 | 17.2±1.2 | 15.01±0.14 | 0.24 | 3 |
| GDS12-4 | 20 | 0.210 | 175 | 2.293 | 1913 | 98.0 | 1.190 | 2823 | 19.0±1.6 | 14.46±0.89 | 1.54 | 3 |
| GDS12-5 | 20 | 0.172 | 205 | 1.979 | 2364 | 99.8 | 1.185 | 2811 | 17.9±1.4 | 14.62±1.08 | 1.52 | 2 |
| GDS12-6 | 20 | 0.149 | 284 | 1.795 | 3424 | 98.9 | 1.180 | 2799 | 17.0±1.1 | 14.80±0.30 | 1.25 | 17 |
| GDS12-7 | 20 | 0.215 | 222 | 2.380 | 2453 | 93.3 | 1.174 | 2786 | 18.5±1.4 | 13.81±0.53 | 1.51 | 8 |
|  |  |  |  |  |  |  |  |  |  |  |  |  |
| 8424-20F* | 20 | 0.242 | 133 | 1.779 | 979 | 99.8 | 1.159 | 2749 | 27.4±2.6 | 15.03± 0.25 | 1.13 | 21 |

Track densities (ρ) are ×10^6^ tracks cm^-2^. All analyses are by the External Detector Method using 0.5 for the 4π/2π geometry correction factor. Apatite ages calculated using dosimeter glass CN5 and zeta-612 = 348.8±5.5 (±1σ) by Ganqing Xu. P(χ^2^) is the probability of obtaining χ^2^ value for v degrees of freedom (where v is the number of crystals -1) (Galbraith, 1981); pooled ρs/ρi ratio is used to calculate age and uncertainty where P(χ^2^) >5%; mean ρs/ρi ratio is reported for samples where P(χ^2^) <5% and for which Central ages (Galbraith and Green, 1990) are calculated.

**SI*2-2* U-Pb Analytical methods and results**

***Analytical methods***

Measurements of U, Th and Pb isotopes were conducted using a Cameca IMS-1280 SIMS at the Institute of Geology and Geophysics, Chinese Academy of Sciences in Beijing. The instrument description and analytical procedure can be found in Li et al. (2009). The primary O^2–^ ion beam spot was about 20-30 micron in diameter. Positive secondary ions were extracted with a 10 KV potential. Oxygen flooding was used to increase the O_2_ pressure to ca. 5×10^-6^ Torr in the sample chamber, enhancing the secondary Pb^+^ sensitivity to a value of 25 cps/nA/ppm for zircon. In the secondary ion beam optics, a 60 eV energy window was used, together with a mass resolution of ca. 5400 (at 10% peak height) to separate Pb^+^ peaks from isobaric interferences. A single electron multiplier was used in ion-counting mode to measure secondary ion beam intensities by peak jumping mode. Analyses of the standard zircon TEMORA 2 were interspersed with unknown grains. Each measurement consists of 7 cycles. Pb/U calibration was performed relative to zircon standard TEMORA 2 (^206^Pb/^238^U age = 417 Ma, Black et al., 2004); U and Th concentrations were calibrated against zircon standard 91500 (Th = 29 ppm, and U = 81 ppm, Wiedenbeck et al., 1995). A long-term uncertainty of 1.5% (1 RSD) for ^206^Pb/^238^U measurements of the standard zircons was propagated to the unknowns (Li et al., 2010), despite that the measured ^206^Pb/^238^U error in a specific session is generally 1% (1 RSD). Measured compositions were corrected for common Pb using non-radiogenic ^204^Pb. Corrections are sufficiently small to be insensitive to the choice of common Pb composition, and an average of present-day crustal composition (Stacey and Kramers, 1975) is used for the common Pb assuming that the common Pb is largely surface contamination introduced during sample preparation. Data reduction was carried out using the Isoplot/Ex v. 2.49 program (Ludwig, 2001). Uncertainties on individual analyses in data tables are reported at 1σ level; Concordia U-Pb ages are quoted with 95% confidence interval, except where noted otherwise.

In order to monitor the external uncertainties of SIMS U-Pb zircon dating calibrated against TEMORA 2 standard, an in-house zircon standard Qinghu was alternately analyzed as an unknown together with other unknown zircons. Six measurements on Qinghu zircon (see Table S2-2-1) yield a Concordia age of 159.3 ± 1.9 Ma, which is identical within error with the recommended value of 159.5 ± 0.2 Ma (Li et al. 2013).

***Zircon U–Pb results***

Zircons from the sample are mostly long columnar or acicular in shape. The grains range from 100-200 microns in lengths and have length to width ratios between 2:1 and 3:1. Most zircons in the sample are pale yellow or colourless. CL images of zircons show fine oscillatory zoning and irregular cores. Twenty-six laser spots were conducted on 26 zircon grains from this sample. All analyzed zircons have Th content of 58-3788 ppm and U content of 115-2729 ppm. The 26 zircon grains dated have an average Th/U ratio of 0.719 (see Table S2-2-1). On the concordia diagram, most analyses are concordant or nearly concordant and yield ^206^Pb/^238^U ages ranging between 24 and 21 Ma (see Figure S2-2-1) with concordia age of 22.34 ± 0.22 Ma (MSWD = 1.07).

***References***

1. Black LP, Kamo SL, Allen CM, Davis DW, Aleinikoff JN, Valley JW, Mundil R, Campbell IH, Korsch RJ, Williams IS, Foudoulis C(2004), Improved ^206^Pb/^238^U microprobe geochronology by the monitoring of a trace-element-related matrix effect; SHRIMP, ID-TIMS, ELA-ICP-MS and oxygen isotope documentation for a series of zircon standards. Chemical Geology, 205: 115-140.
2. Li XH, Liu Y, Li QL, Guo CH, Chamberlain KR (2009), Precise determination of Phanerozoic zircon Pb/Pb age by multi-collector SIMS without external standardization.Geochem.GeophysGeosyst10, Q04010, doi:10.1029/2009GC002400.
3. Li QL, Li XH, Liu Y, Tang GQ, Yang JH, Zhu WG (2010) Precise U-Pb and Pb-Pb dating of Phanerozoicbaddeleyite by SIMS with oxygen flooding technique. J AnalAtSpectrom 25: 1107-1113.
4. Li XH, Tang GQ, Gong B, Yang YH, Hou KJ, Hu ZC, Li QL, Liu Y,Li WX (2013)Qinghu zircon: A working reference for microbeam analysis of U-Pbage and Hf and O isotopes. Chin Sci Bull 58: 4647-4654.
5. Ludwig KR (2001)Users manual for Isoplot/Ex rev. 2.49.Berkeley Geochronology Centre Special Publicationb 1, 56p.
6. Stacey JS, Kramers JD (1975) Approximation of terrestrial lead isotope evolution by a two-stage model. Earth and Planetary Science Letters 26: 207-221.
7. Wiedenbeck M, Alle P, Corfu F, Griffin WL, Meier M,Oberli F, Quadt A V, Roddick JC, Spiegel W(1995) Three natural zircon standards for U-TH-PB, LU-HF, trace element and REE analyses. Geostandards Newsletter, 19: 1–23. doi: 10.1111/j.1751-908X.1995.tb00147.x.

**
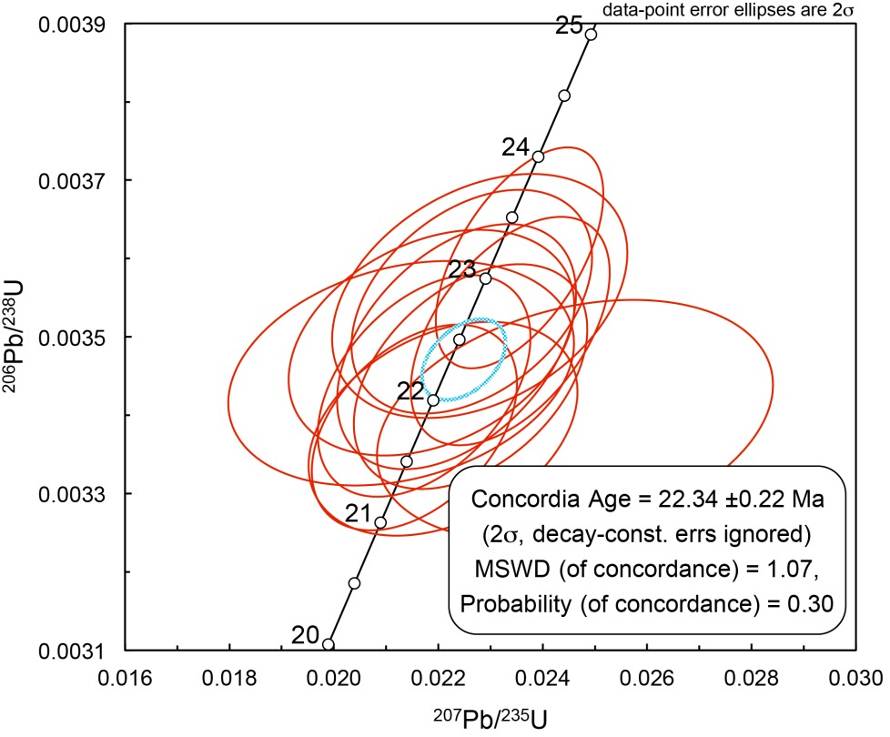
**

Figure SI2-2-1 ^206^Pb/^238^U concordia diagram

Table SI2-2-1 Results of U, Th and Pb isotopes Measurements.

| Spot No. | Content | | Th/U | Isotopic ratio | | | | | | Age/Ma | | | |
| --- | --- | --- | --- | --- | --- | --- | --- | --- | --- | --- | --- | --- | --- |
|  | Th | U |  | ^207^Pb/^206^Pb | 1 σ | ^207^Pb/^235^U | 1 σ | ^206^Pb/^238^U | 1 σ | ^207^Pb/^235^U | 1 σ | ^206^Pb/^238^U | 1 σ |
| 13-1 01 | 642 | 987 | 0.650 | 0.04654 | 4.74 | 0.02279 | 5.07 | 0.0036 | 1.79 | 22.9 | 1.1 | 22.9 | 0.4 |
| 13-1 02 | 723 | 1089 | 0.664 | 0.04648 | 3.76 | 0.02235 | 4.18 | 0.0035 | 1.83 | 22.4 | 0.9 | 22.4 | 0.4 |
| 13-1 03 | 933 | 1511 | 0.618 | 0.04542 | 4.82 | 0.02187 | 5.11 | 0.0035 | 1.68 | 22.0 | 1.1 | 22.5 | 0.4 |
| 13-1 04 | 380 | 774 | 0.490 | 0.05261 | 6.02 | 0.02462 | 6.29 | 0.0034 | 1.85 | 24.7 | 1.5 | 21.8 | 0.4 |
| 13-1 05 | 402 | 799 | 0.503 | 0.04736 | 4.47 | 0.02209 | 4.76 | 0.0034 | 1.65 | 22.2 | 1.0 | 21.8 | 0.4 |
| 13-1 06 | 832 | 1411 | 0.589 | 0.04617 | 3.37 | 0.02154 | 3.73 | 0.0034 | 1.58 | 21.6 | 0.8 | 21.8 | 0.3 |
| 13-1 07 | 905 | 1300 | 0.696 | 0.04585 | 3.44 | 0.02173 | 3.83 | 0.0034 | 1.68 | 21.8 | 0.8 | 22.1 | 0.4 |
| 13-1 08 | 1069 | 1241 | 0.862 | 0.04889 | 3.38 | 0.02129 | 6.35 | 0.0035 | 1.69 | 21.4 | 1.3 | 22.2 | 0.4 |
| 13-1 09 | 604 | 1087 | 0.556 | 0.04756 | 3.66 | 0.02262 | 4.04 | 0.0034 | 1.70 | 22.7 | 0.9 | 22.2 | 0.4 |
| 13-1 10 | 3788 | 2729 | 1.388 | 0.04745 | 2.28 | 0.02356 | 2.78 | 0.0036 | 1.60 | 23.6 | 0.7 | 23.2 | 0.4 |
| 13-1 11 | 520 | 1112 | 0.467 | 0.04777 | 3.44 | 0.02517 | 3.84 | 0.0038 | 1.70 | 25.2 | 1.0 | 24.6 | 0.4 |
| 13-1 12 | 1741 | 1736 | 1.003 | 0.04834 | 2.86 | 0.02338 | 3.33 | 0.0035 | 1.70 | 23.5 | 0.8 | 22.6 | 0.4 |
| 13-1 13 | 719 | 998 | 0.720 | 0.04632 | 3.82 | 0.02264 | 4.16 | 0.0035 | 1.65 | 22.7 | 0.9 | 22.8 | 0.4 |
| 13-1 14 | 577 | 1189 | 0.485 | 0.04805 | 3.47 | 0.02186 | 4.99 | 0.0034 | 1.82 | 22.0 | 1.1 | 22.1 | 0.4 |
| 13-1 15 | 1953 | 1798 | 1.086 | 0.04823 | 3.10 | 0.02370 | 3.52 | 0.0036 | 1.67 | 23.8 | 0.8 | 22.9 | 0.4 |
| 13-1 16 | 465 | 929 | 0.501 | 0.04836 | 3.95 | 0.02282 | 4.33 | 0.0034 | 1.78 | 22.9 | 1.0 | 22.0 | 0.4 |
| 13-1 17 | 1153 | 1486 | 0.776 | 0.04488 | 3.27 | 0.02290 | 3.71 | 0.0037 | 1.75 | 23.0 | 0.8 | 23.8 | 0.4 |
| 13-1 18 | 686 | 1085 | 0.632 | 0.04733 | 3.60 | 0.02263 | 4.13 | 0.0035 | 2.02 | 22.7 | 0.9 | 22.3 | 0.4 |
| 13-1 19 | 1141 | 1437 | 0.794 | 0.04725 | 3.09 | 0.02318 | 3.52 | 0.0036 | 1.68 | 23.3 | 0.8 | 22.9 | 0.4 |
| 13-1 20 | 936 | 1180 | 0.793 | 0.04966 | 3.33 | 0.02406 | 3.74 | 0.0035 | 1.69 | 24.1 | 0.9 | 22.6 | 0.4 |
| 13-1 21 | 474 | 752 | 0.630 | 0.04941 | 4.20 | 0.02357 | 4.58 | 0.0035 | 1.84 | 23.7 | 1.1 | 22.3 | 0.4 |
| 13-1 22 | 664 | 997 | 0.666 | 0.04570 | 3.85 | 0.02199 | 4.23 | 0.0035 | 1.74 | 22.1 | 0.9 | 22.5 | 0.4 |
| 13-1 23 | 781 | 1222 | 0.639 | 0.04550 | 4.37 | 0.02224 | 4.69 | 0.0035 | 1.71 | 22.3 | 1.0 | 22.8 | 0.4 |
| 13-1 24 | 1111 | 1364 | 0.815 | 0.04855 | 3.08 | 0.02247 | 4.23 | 0.0035 | 1.66 | 22.6 | 0.9 | 22.3 | 0.4 |
| 13-1 25 | 1154 | 1357 | 0.851 | 0.04693 | 3.90 | 0.02357 | 4.25 | 0.0036 | 1.69 | 23.7 | 1.0 | 23.4 | 0.4 |
| 13-1 26 | 439 | 765 | 0.573 | 0.04457 | 4.85 | 0.02100 | 5.14 | 0.0034 | 1.70 | 21.1 | 1.1 | 22.0 | 0.4 |

**SI*2-3*** **Results of** **age dating of radiolarian fossils**


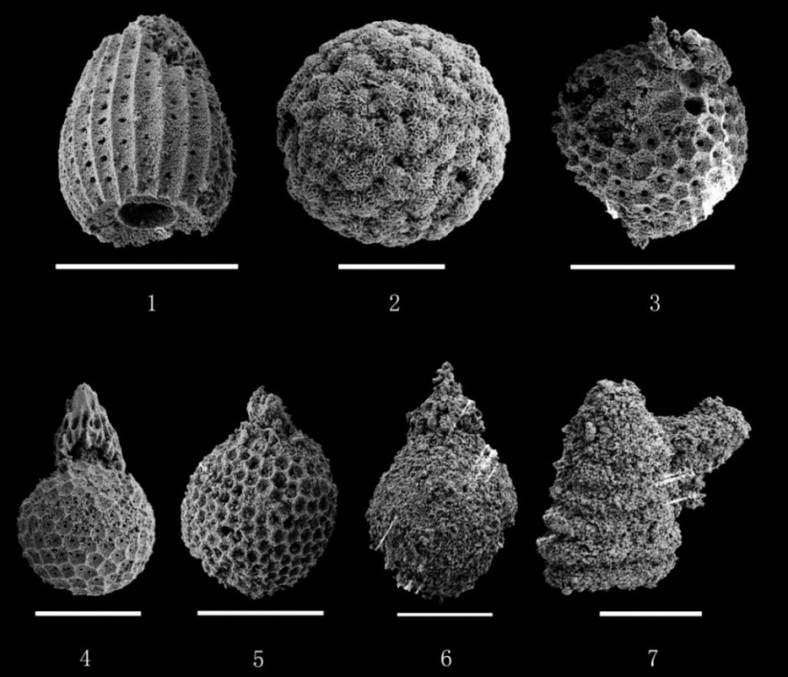


a: Age ranges of radiolarian fossils from purple-coloured chert pebbles sampled from the Kailas conglomerate in the area 10 km northwest of Xigaze (Ages determined by the Nanjing Geology and Paleontology Research Institute, Chinese Academy of Sciences).

*1. Thanarlabrouweri* Late Tithonian (Late Jurassic) to Cenomanian (Late Cretaceous).

2. *Acaeniotyliae(?)* Late Tithonian to Cenomanian.

3. *Cryptamphorella* sp. cf. *C*. *conara* (Foreman) Late Tithonian to Maastrichtian (Late Cretaceous).

4, 5. *Hiscocapsa (?)*sp.

6. *Hiscocapsa(?)* sp. Bathonian (Middle Jurassic) to Albian (Early Cretaceous).

7. *Nassellaria* -- Bathonian to Albian


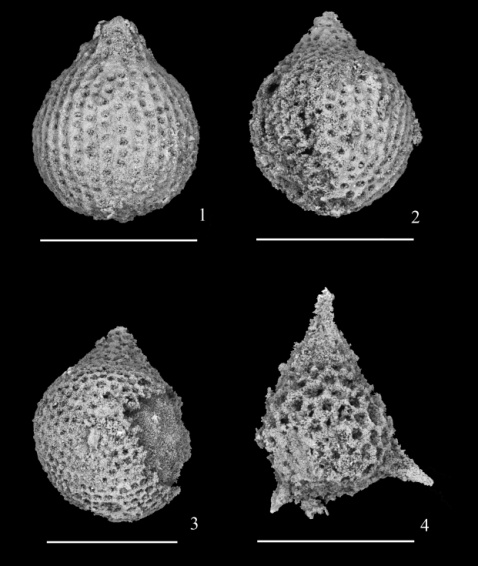


b: Age ranges of radiolarian fossils extracted from purple-coloured chert within the ophiolitic suite of the Zhangbo suture zone forming the hanging wall of the GCT at Langxian, 10 km south of Xigaze, (Ages determined by the Nanjing Geology and Paleontology Research Institute, Chinese Academy of Sciences). Note: most of the radiolarian fossils extracted from this chert could not be identified due to their poor preservation state, but those that could be identified are of Middle Jurassic age.

1-2. *Striatojaponocapsasynconexa* [O’Dogherty, Gorican and Dumitrica, 2006] (Late Bajocian to early Bathonian, Middle Jurassic).

3. *Stichocapsarobusta* [Matsuoka 1984] (Latest Bajocian to Early Callovian, Middle Jurassic).

4. *Sethocapsa* sp.
